# A Stereospecific Synthesis and In Vitro Anti-Influenza H1N1 Properties of Lithocholic Acid-Based *Spiro*-1,2,4-trioxolane

**DOI:** 10.3390/molecules30234613

**Published:** 2025-12-01

**Authors:** Irina Smirnova, Alexander Lobov, Liana Zakirova, Dmitriy Polovyanenko, Irina Bagryanskaya, Vladimir Zarubaev, Oxana Kazakova

**Affiliations:** 1 Ufa Institute of Chemistry, Ufa Federal Research Centre, Russian Academy of Sciences, 71, pr. Oktyabrya, 450054 Ufa, Russia; si8081@yandex.ru (I.S.); lobovan@anrb.ru (A.L.); liana.zakirova@gmail.com (L.Z.); 2 N. N. Vorozhtsov Novosibirsk Institute of Organic Chemistry, Siberian Branch of Russian Academy of Sciences (SB RAS), 630090 Novosibirsk, Russia; dpolo@nioch.nsc.ru (D.P.); bagryan@nioch.nsc.ru (I.B.); 3 Experimental Virology Laboratory, Department of Virology, St. Petersburg Pasteur Institute of Epidemiology and Microbiology, 14 Mira St., 197001 St. Petersburg, Russia; zarubaev@gmail.com

**Keywords:** bile acids, lithocholic acid, 1,2,4-trioxolane, Griesbaum co-ozonolysis, X-ray analysis, ADME, PASS prediction, anti-viral

## Abstract

Bile acids provide a versatile platform for the design of biologically active compounds due to their amphiphilic structure, biocompatibility, and capacity for diverse chemical modifications. Among them, lithocholic acid is a promising scaffold for designing and revealing new antiviral agents. A novel lithocholic acid-based 3-*spiro*-1,2,4-trioxolane was synthesized by Griesbaum co-ozonolysis of methyl 3-*O*-methyl-oximino-lithocholate and 4-(trifluoromethyl)-cyclohexanone, and its structure was confirmed by 2D NMR and X-ray crystallographic analysis. Lithocholic acid derivatives were evaluated for cytotoxicity and anti-influenza activity against A/Puerto Rico/8/34 (H1N1), showing that steroid 1,2,4-trioxolane **3** exhibited the highest potency (IC_50_ 4.3 µM, SI 11) compared to the parent methyl-3-oxo-lithocholate **1** (IC_50_ > 84 µM, SI 1). In silico ADME predictions revealed several favorable drug-like properties, including a highly three-dimensional structure (Fsp^3^ = 0.97), significant lipophilicity (LogP = 7.54), and the presence of key pharmacophores such as a peroxide moiety and a trifluoromethyl group. Taken together, a stereospecific synthesis of a lithocholic acid 3-*spiro*-1,2,4-trioxolane by Griesbaum co-ozonolysis was realized and the first evidence of anti-influenza activity in the steroid-1,2,4-trioxolane series was found.

## 1. Introduction

The steroid system has been extensively used and developed as a privileged scaffold with diversified medicinal properties in the drug discovery process. A striking example of this application is a steroid polyamine squalamine that became the first representative of a previously unknown class of natural antibiotics of animal origin [1]. Among steroid molecules, bile acids have indeed emerged as highly useful scaffolds for the development of conjugates with various biologically active molecules. Such conjugates exhibit improved water solubility, increased metabolic stability, and, in some cases, selective cellular penetration and a broad spectrum of activities [2]. For example, the bile acid conjugates with amino acids or peptides which inhibited HIV-1 replication at a concentration as low as 0.02 µg/mL have analgesic and anticancer activities [3].

Lithocholic acid (LCA) is formed through the microbial metabolism of primary bile acids in the human intestine [4] and has recently gained renewed interest for immunomodulatory, anti-inflammatory, and anticancer effects [5]. Notably, emerging evidence indicates that LCA and its semisynthetic derivatives possess antiviral potential, offering a new avenue in the development of antiviral therapies. LCA is known to interact with several nuclear and membrane-bound receptors, such as the pregnane X receptor, vitamin D receptor, and G-protein-coupled bile acid receptor, which are involved in modulating host innate immunity and inflammatory responses [6]. This ability to influence host signaling pathways may play a critical role in restricting viral replication and enhancing antiviral defense. In vitro studies have demonstrated that LCA and chenodeoxycholic acid at physiological concentrations can suppress the replication of porcine delta-coronavirus, via the activation of GPCR signaling, leading to the upregulation of interferon lambda-3 and interferon-stimulated gene 15 [7]. This approach aims to improve the characteristics of LCA such as bioavailability, lower toxicity, and more efficient drug delivery [8]. Lithocholic acid oleate has been shown to upregulate the expression of human beta-defensin 1 and cathelicidin LL-37 in human keratinocyte cells [9]. The molecular hybridization of dihydroartemisinin and ursodeoxycholic acid resulted in a significant reduction in the viral replication of SARS-CoV-2 [10]. Bile acids play a vital role in maintaining intestinal homeostasis and combating infections; for example, deoxycholic acid can restore interferon type I responses to restrict the Chikungunya virus [11]. A recent study showed that a combination of LCA and LCA-oleate provided preventive antiviral protection against herpes simplex virus type 1 in both in vitro and in vivo models [12]. We have shown that the 3-oxo-lithocholic acid Mannich base connected with *N*-methylpiperazine increased antiviral activity against the Flu A (H1N1) virus along with a two-fold reduction in toxicity in MDCK cells [13].

On the other hand, there is a large group of natural and semisynthetic compounds with peroxide units that exhibit a variety of biological activity, including anthelmintic, antiprotozoal, fungicidal, antiviral, and others [14,15]. The most known natural peroxide is artemisinin with high antimalarial activity, the discovery of which by Chinese pharmaceutical chemist Tu Youyou was awarded the Nobel Prize in Physiology or Medicine in 2015. Chinese scientists also provided the first hint that artemisinin might have antiviral activity [16]. The antiviral activity of artemisinin and its semisynthetic derivative artesunate includes the inhibition of viruses, such as human cytomegalovirus and other members of the *Herpesviridae* family (e.g., herpes simplex virus type 1 and Epstein–Barr virus), hepatitis B virus, hepatitis C virus, and bovine viral diarrhea virus [17]. The broad anti-cytomegaloviral activity of artesunate was developed against viral strains and therapy-resistant mutants, and it was shown that viral replication is blocked at a very early stage and that in vitro efficacy is optimized when artesunate was applied as fractional doses with a synergistic effect with the artesunate–maribavir combination treatment [18]. Artesunate showed an inhibitive effect on HCV replication and may be a novel supplemental co-therapy with interferon-2b and ribavirin for HCV and as an alternative strategy to combat resistance mechanisms that have emerged in the presence of direct antiviral agents [19]. Recently, artesunic acid–quinoline hybrids demonstrated an inhibitory activity against SARS-CoV-2 in vitro (EC_50_ 13–19 μM) and no cytotoxic effects on VeroE6 cells (CC_50_ up to 110 μM) [20]. The case of artemisinin could be supported by other examples of the fairly common practice for drugs with known activity to be tested for other types of effects. For instance, amodiaquine, being 4-aminoquinoline and related to chloroquine with antimalarial activity, was recently found to be an effective inhibitor of the viral entry of SARS-CoV-2 in vitro and in vivo [21] and completed Phase 2 trials for COVID-19 treatment in South Africa (NCT04532931).

Besides artemisinin, more examples of natural compounds holding a peroxide group with antiviral activity could be named. Norsesterterpene peroxide acid muqubilone, isolated from the Red Sea sponge *Diacarnus erythraeanus*, showed in vitro antiviral activity against herpes simplex type 1 with ED_50_ value of 7.5 μg/mL [22]. A series of cholesterol and β-sitosterol derivatives with a peroxide bridge at the B-ring were synthesized and screened for their anti-HBV activity; among them, 5α,8α-cyclicobioxygen-6-vinyl-3-oxo-cholesterone exhibited high inhibitory capacity against HBsAg and HBeAg secretion in HepG2.2.15 cells, whereas nearly no cytotoxicity on HepG2.2.15 cells at 3.13 μg/mL was observed, showing good therapeutic index [23]. Ergosterol peroxide inhibited Porcine delta-coronavirus infection and regulated host immune responses by downregulating the activation of the NF-κB and p38/MAPK signaling pathways in vitro, which makes it a potential candidate for the development of a new anti-PDCoV therapy [24]. Ergosterol peroxide also suppressed IAV-associated inflammation and apoptosis via blocking RIG-I signaling, which may serve as a supplementary approach to the treatment of influenza [25].

Advances in the development of natural peroxides prompted the synthesis of steroid-based 1,2,4,5-tetraoxanes [26] (including dimer molecules [27]), as well as 1,2,4-trioxanes [28], that demonstrated anti-malarial, antiproliferative, antimycobacterial [29], and other types of activities.

Another sub-class of peroxides, namely 1,2,4-trioxolanes, prepared via the Griesbaum co-ozonolysis, were firstly developed on the basis of the adamantanone scaffold affording an extensive series named as “OZ” [30,31]. One of them, ozonide OZ277, also known as arterolane maleate, was approved for marketing in India as a combination product with piperaquine phosphate (Synriam) to treat malaria. Another adamantanone-based 1,2,4-trioxolane artefenomel (OZ439) was until very recently a leading candidate to replace the artemisinin and its semisynthetic analogs in artemisinin combination therapy for uncomplicated malaria [32].

The Griesbaum co-ozonolysis [33], the last three decade anniversary of which was celebrated in 2025, employs *O*-methyl oximes and carbonyl compounds as substrates to produce 1,2,4-trioxolanes using ozone. Compared to conventional olefin ozonolysis, this method offers several significant advantages such as improved selectivity, preparation of unsymmetrically substituted ozonides without the formation of undesired by-products, etc. [34].

The group of steroid-based *spiro*-1,2,4-trioxolanes is a new and few in number set of hybride molecules first launched in 2014 [35], then expanded to deoxycholic acid 3′-trifluoromethylated 1,2,4-trioxolanes, which demonstrated in vitro antimalarial activity against the chloroquine-sensitive T96 and chloroquine-resistant K1 strains of *Plasmodium falciparum* [36].

According to all previous information, this article’s concern is the synthesis of a new steroid-based 3-*spiro*-1,2,4-trioxolane molecule, an in silico ADMET study, and the evaluation of in vitro anti-influenza activity.

## 2. Results and Discussion

### 2.1. Chemistry

**Scheme 1:**
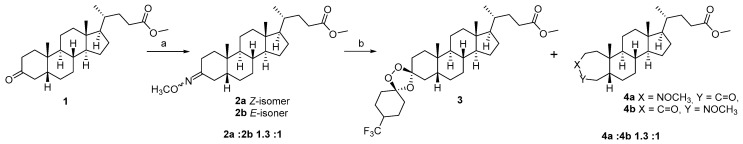
Reagents and conditions: (a) MeONH_2_·HCl, MeOH–C_5_H_5_N, 115 °C; (b) O_3_, 4-(trifluoromethyl)–cyclohexanone, CH_2_Cl_2_–cyclohexane, 0 °C.

To prepare the target hybrid molecule holding the lithocholic acid scaffold and 1,2,4-trioxolane unit at C3 position, a synthetic strategy involving the abovementioned Griesbaum co-ozonolysis (Scheme 1) was selected. As the starting material, 3-oxo-lithocholic acid methyl ester **1** was obtained via a standard procedure [35] from commercially available LCA. The subsequent reaction of compound **1** with methoxy-hydroxylamine hydrochloride under reflux in a methanol–pyridine mixture afforded the *O*-methyl-ketoxime **2** in a yield of 93%. In the ^13^C NMR spectrum of compound **2**, a pronounced downfield chemical shift in the C3 carbon signal was observed, along with the appearance of a characteristic signal corresponding to the C=NOCH_3_ moiety at δ 165 ppm, indicating the full conversion of the 3-oxo-group. Additionally, the ^1^H NMR spectrum revealed signals of a methoxy-group in the region of δ 3.9–4.0 ppm. The intensity ratio of the methoxy proton signals in the ^1^H NMR spectrum, together with the presence of duplicated C3 carbon signals in the ^13^C NMR spectrum, supports the formation of a mixture of two Z/*E* isomers of the *O*-methyl-ketoxime **2a** and **2b** (see Supplementary Materials Figures S1–S10).

As a carbonyl component in the Griesbaum co-ozonolysis reaction, 4-trifluoromethylcyclohexanone has been used, which, to the best of our knowledge, was not previously involved in such transformations despite the considerable attention to other substituted cyclohexanones. The selection of 4-trifluoromethylcyclohexanone as the ketone component for the Griesbaum co-ozonolysis was guided by well-established stereochemical trends for 4-substituted cyclohexanones in this reaction. In particular, the application of 4-alkyl- and 4-aryl-substituted cyclohexanones (such as 4-methyl- and 4-phenylcyclohexanones) has been previously described [31,36], affording the formation of the stable tetrasubstituted trioxolanes with the ratio diastereomers from 2.5:1 to 20:1. Tang and co-authors have noted that diastereoselectivity is a function of the nature and size of the substituents at the C4 position of the cyclohexanone ring. The bulky substituents at the C4 position strongly bias the approach of the carbonyl oxide, favoring axial attack and thus promoting the formation of a single dominant diastereomer [31]. This steric steering effect is even more pronounced for highly electron-withdrawing and volumetric groups (such as CF_3_ in our case), which stabilize the preferred transition state which provides steric shielding around the emerging endoperoxide bond, contributing to the increased stability of the resulting 1,2,4-trioxolane, and suppress the formation of alternative stereoisomers [37]. Successful applications of other CF_3_-containing ketones in related trioxolane syntheses, including steroid-based frameworks [35], further support the suitability of 4-trifluoromethylcyclohexanone for constructing structurally defined and stable peroxide derivatives. The use of other substituted cyclohexanones as the carbonyl component revealed certain drawbacks; for example, the reaction was proceeded with low regio- and stereoselectivity, leading to the formation of complex mixtures of isomers rather than a single dominant product [35,36,38,39].

So, the last stage of our approach involved the interaction of *O*-methyl-ketoxime **2** and 4-(trifluoromethyl)-cyclohexanone under ozone in a mixture of methylene chloride and cyclohexane at 0 °C. The reaction was monitored by TLC (using the solvent system chloroform–ethyl acetate, 40:1); after finishing, the solvent was evaporated and the formation of several products in the crude reaction was observed. After purification by column chromatography, 3-*spiro*-1′,2′,4′-trioxolane **3** as the main product (yield 72%) and a mixture of isomeric *O*-methoxylactams **4a** and **4b** (total yield 24%) were isolated. In contrast to the previously described spiro-trioxolanes based on deoxycholic acid and triterpenoids [35,36,38,39], in this case the use of 4-trifluoromethylcyclohexanone in the Griesbaum co-ozonolysis led to the selective formation of a single (3*S*)-diastereomer **3** in a quantitative yield, so we can conclude that the use of 4-(trifluoromethyl)-cyclohexanone allowed a stereospecific synthesis of steroid 1′,2′,4′-trioxolane to be realized.

**Figure 1:**
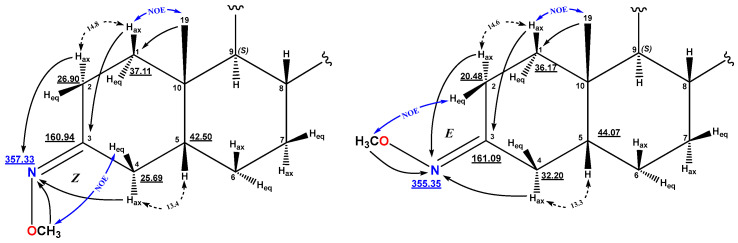
The key NMR NOESY assignments of compounds **2a**—*Z* (**left**) and **2b**—*E* (**right**).

In order to confirm the structure and stereochemistry of the obtained compounds, COSY, NOESY, HSQC, and HMBC experiments were used. According to the NMR spectra, compound **2** is presented as a mixture of two *Z/E* isomers relative to the imine bond in a ratio of 1.3 (**2a**) to 1 (**2b**). Protons and carbon and nitrogen atoms, part of the A and B rings, are represented by a double set of signals in the ^1^H, ^13^C, and ^15^N NMR spectra. The greatest differences in the chemical shifts in the signals between the *Z* and *E* isomers are observed for the positions closest to the isomerism site: C2, C3, C4, and NOCH_3_. The assignment of methyl oximes to the Z/E isomers was made on the basis of NOESY spectral data. For the major isomer, a NOESY cross peak was observed between the signals of the methyl oxime group protons and the equatorial proton at position C4 (δ_H_ 3.79/2.79 ppm), indicating the *Z*-isomer **2a**. The *E*-configuration of the imine bond in the minor isomer was established based on the observed NOESY cross peak between NOCH_3_ and Heq-2 (δ_H_ 3.78/2.95 ppm) (Figure 1).

**Figure 2:**
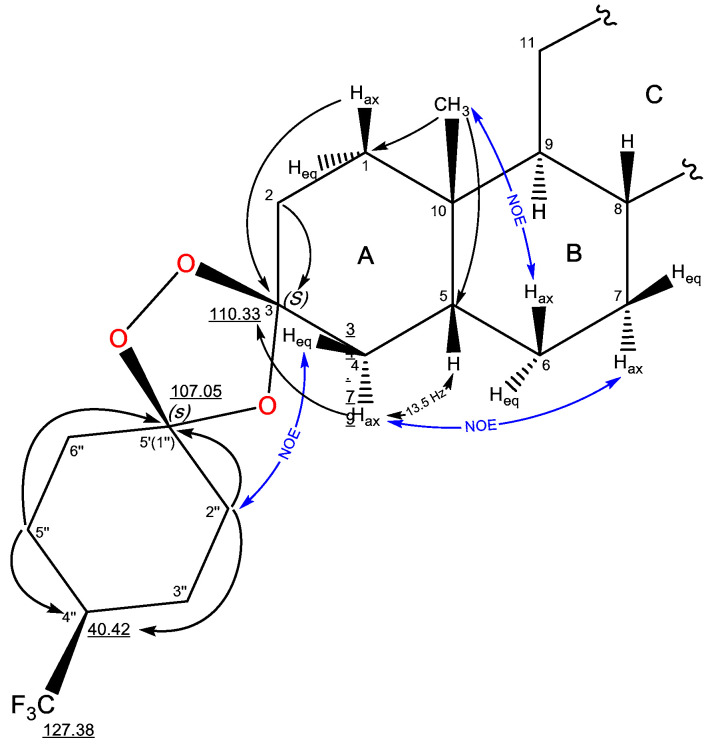
NMR signal assignments and significant correlations of compound **3**.

The structure of compound **3** was established by analysis of ^1^H, ^13^C, and ^19^F NMR spectra by using two-dimensional correlation {^1^H, ^13^C} HSQC and HMBC, {^19^F, ^1^H} HETCOR, as well as {^1^H, ^1^H} COSY-DQF and NOESY (for NMR data of compound **3** see Supplementary Materials Figures S11–S22). The formation of the 1′,2′,4′-trioxolane ring is confirmed by the signals of the *spiro* centers with characteristic δ_C_ values of 107.05 and 110.33 ppm. The expected presence of the peroxidic carbon atom at position C3 of the A-ring was established based on the HMBC cross peaks of the protons of H_ax_-1 (δ_H_ 1.21 ppm), H_ax_-4, and H_eq_-4 (δ_H_ 2.15 and 1.51 ppm) with a signal at δ_C_ 110.33 ppm. For the second acetal carbon at δ_C_ 107.05 ppm, HMBC correlations with the methylene protons of the 4-CF_3_-cyclohexane ring are observed. The presence of the trifluoromethyl function at position 4″ of the *spiro*-fused cyclohexane results in a quartet splitting of the C4″ signal (δ_C_ 40.42 ppm) with *^2^J*_CF_ = 27.4 Hz and broadening of the C3″ and C5″ signals in the ^13^C{^1^H} spectrum. In the ^19^F spectrum, the CF_3_ group signal (δ_F_ −73.26 ppm) is represented by a doublet with coupling constant ^3^*J*_HF_ = 8.1 Hz on the methine proton H-4″ (δ_H_ 2.03 ppm), which is confirmed in the {^19^F, ^1^H} HETCOR spectrum. The main correlations are presented in Figure 2.

**Figure 3:**
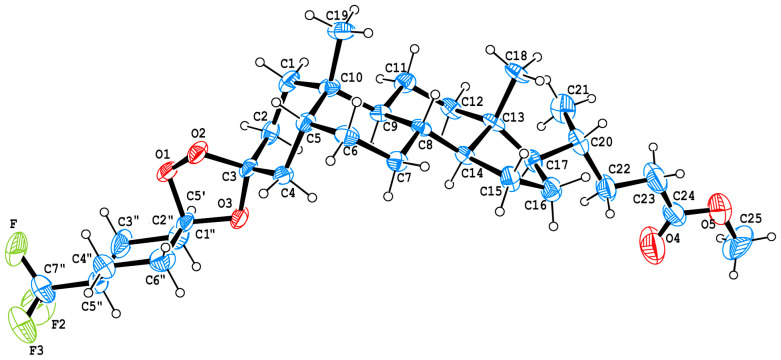
Molecular structure of methyl 3(*S*)-3,5′-dispiro-[(4′′-trifluoromethyl-cyclohexyl)-1′,2′,4′-trioxolane]-5β-cholan-24-oate **3**. ORTEP drawing of compound **3** showing thermal ellipsoids at the 40% probability level.

X-ray crystallography is crucial in pharmaceutical research for newly synthesized compounds, providing absolute configuration determination and revealing hydrogen bonding, π-π stacking, and other interactions that influence stability and biological activity [40]. Single crystals of methyl (3*S*)-3,5′-dispiro-[(4′′-trifluoromethyl-cyclohexyl)-1′,2′,4′-trioxolane]-5*β*-cholan-24-oate **3** suitable for structural analysis were successfully grown through slow evaporation from diethyl ether. The obtained crystal structures were analyzed using PLATON (v2.0) and MERCURY (v2.0) programs [41,42]. The structure of steroid trioxolane **3** is formed by two crystalographically independent molecules, one of which is shown in Figure 3. For these molecules, the bond lengths and bond angles are very close, and the same for the statistical means [43]. All cyclohexane fragments have a “chair” conformation; the 5-membered cycles have a “twist” conformation. Note that no reduced intermolecular contacts are observed in the crystal packing of compound **3.**

The 1,2,4-trioxolane fragment contains O-C-O-O atoms characterized by the anomeric effect [44]. Therefore, the lengths of O3-C3, O2-C3, O3-C5′, and O3-C5′ bonds differ from the standard value (1.43 Å [43]) and are 1.438 (9), 1.439 (8), 1.408 (10), and 1.443 (9) Å, respectively. For similar bonds in the previously studied deoxycholic acid 3-*spiro*-5′-(3′-methyl-3′-trifluoromethyl-1,2,4-trioxolane) (compound **3a** see Supplementary Materials Figure S23), the lengths are 1.434 (3), 1.433 (3), 1.421 (4), and 1.411 (3) Å. The peroxide bond lengths in both these trioxolanes are 1.461 (compound **3**) and 1.473 (for compound **3a**, Figure S23) which correspond to standard values [44].

The O2-C3-O3 bond angles (103.41 (compound **3**) and 102.45 (compound **3a**, Figure S23) are similar in value, while the O1-C5′-O3 bond angle at C5′ differs significantly (102.95 (compound **3**) and 106.19 (compound **3a**, Figure S23)). This is due to the fact that in compound **3** it is a part of the cyclohexane fragment and serves as a *spiro*-center. Bond angles O2-O1-C5′ 102.02 and 101.92, as well as C5′-O3-C3 107.27 and 107.43 (which are related to compound **3** and compound **3a**, Figure S23) are close in values, which indicates the similarity of the conformational state (twist form) of the trioxolane fragments.

According to the NMR spectral data, compound **4** is represented by seven-membered lactams in the form of two isomers at the position of the amide group in a ratio of 1.3 (**4a**) to 1 to 1 (**4b**). The formation of ε-lactams according to the A-ring was established on the basis of the observed carboxamide signals at δ_C_ 171.39 and 171.55 ppm, as well as the δ_N_ values of 195.69 and 192.78 ppm for the methoxy-substituted lactam nitrogens. For the major isomer with the 3-aza-A-homo structure **4a**, HMBC cross peaks of the H-5, H_ax_-2, H_eq_-2, and H_ax_-4a/H_eq_-4a protons with the lactam carbonyl are observed at δ_C_ 171.39 ppm. In addition, in the {^1^H, ^15^N} HMBC spectrum there are cross peaks of the protons of H_ax_-1, H_eq_-1, and H_eq_-4a and the OMe group (δ_H_ 3.73 ppm) with a nitrogen signal at δ_N_ 195.69 ppm. The presence of nitrogen in position C3 is confirmed by the NOESY cross peak of the methoxy substituent with the equatorial proton H_eq_-2 (δ_H_ 3.73/3.54 ppm).

**Figure 4:**
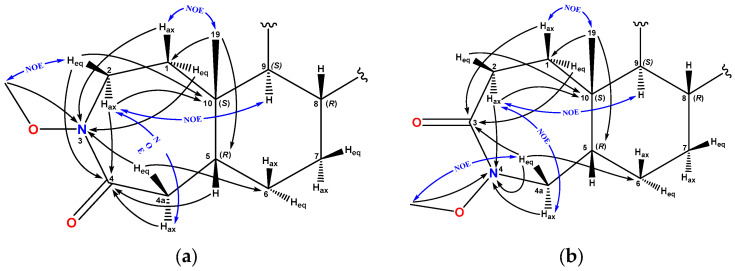
The key NMR NOESY assignments of compounds (**a**)—**4a** and (**b**)—**4b**.

For the minor isomer **4b**, HMBC cross peaks of the H_ax_-1, H_eq_-1, and H_eq_-4a protons with the amide carbonyl are observed at δ_C_ 171.55 ppm and {^1^H, ^15^N} HMBC cross peaks of the protons H_ax_-4a, H_eq_-4a, and H_ax_-2 with δ_N_ 192.78 ppm, confirming the 4-aza-A-homo structure of ring A. For the methoxyl protons of the minor isomer **4b**, NOE interactions with the equatorial proton H_eq_-4a are observed (δ_H_ 3.75/3.20 ppm) (Figure 4 and see Supplementary Materials Figures S24–S33).

Thus, we can conclude that the use of 4-(trifluoromethyl)-cyclohexanone as a carbonyl component in the Griesbaum ozonolysis in the example of LCA provides a one-step stereospecific formation of (*3S*)-stereoisomeric 1,2,4-trioxolane that makes this approach attractive for further development of a new generation of steroidal peroxides.

### 2.2. In Vitro Antiviral Evaluation

Cytotoxicity and anti-influenza properties of the lithocholic acid derivatives **1**–**3** were studied in MDCK cell culture against influenza virus A/PuertoRico/8/34 (H1N1). Oseltamivir carboxylate and rimantadine were used as reference compounds (Table 1). The structure–activity relationship analysis revealed that structural modifications of lithocholic acid markedly affect both cytotoxicity and antiviral potency. The parent methyl lithocholate **1** exhibited low antiviral activity (IC_50_ > 84 µM) and moderate cytotoxicity (CC_50_ 107.4 µM), compared to the reference drug rimantadine. This resulted in a low selectivity index (SI 1). Introduction of a methoxy–oxime substituent at C3 (compound **2**) slightly improved the selectivity (SI 2), possibly due to enhanced polarity and hydrogen bonding capacity, but did not significantly increase antiviral efficacy. The most active derivative was considered to be 1,2,4-trioxolane **3** with IC_50_ 4.3 µM and SI 11, which indicates that the incorporation of a trioxolane ring considerably enhances antiviral activity while maintaining moderate cytotoxicity, but with notably higher antiviral potency and selectivity than rimantadine (CC_50_ 62 µM and IC_50_ 11 µM). Based on the provided data, target lithocholic acid spiro-1,2,4-trioxolane **3** shows higher antiviral activity and selectivity compared to the parent methyl 3-oxo-lithocholate **1** and its methoxy–oxime **2**. While this observation indicates that the introduction of a trioxolane ring positively influences anti-flu activity, this only one example does not allow us to make definitive conclusions regarding the role of the trioxolane moiety as a structural determinant of antiviral potency. Nevertheless, our results suggest that the hybridization of bile acid scaffolds with a 1,2,4-trioxolane unit could produce molecules with antiviral effects.

### 2.3. In Silico ADMET Study and Physicochemical Profiles of Compound ***3***

To assess the pharmaceutical relevance of the synthesized lead molecule **3**, an analysis was conducted across key drug development parameters [45], including physicochemical descriptors, lipophilicity, solubility, pharmacokinetic predictions, and drug-likeness criteria, using SWISS ADME (https://www.swissadme.ch/). The calculated data are presented in Table 2 (for the parent compound **1** see Supplementary Materials Table S2).

### 2.4. Formatting of Mathematical Components

#### 2.4.1. Physicochemical Profile

As one can see from Table 2, compound **3** (C_32_H_49_F_3_O_5_; MW = 570.72 g/mol) is a large and structurally complex molecule with 40 heavy atoms, eight hydrogen bond acceptors, and a high molar refractivity (147.54), indicative of increased polarizability and steric bulk. It displays a pronounced sp^3^ character (fraction Csp^3^ > 0.9), a feature associated with improved clinical success rates due to enhanced three-dimensionality and target specificity [46].

#### 2.4.2. Lipophilicity and Solubility

Lipophilicity, a key determinant of membrane permeability and solubility, was predicted using multiple algorithms. Compound **3** exhibits markedly higher lipophilicity across all models, with a consensus LogP of 7.54, exceeding the optimal range for oral drugs (LogP < 5) [47]. Correspondingly, it is predicted to be poorly soluble, with logS values below −6.5 in all models. Such high lipophilicity and limited solubility may contribute to reduced oral bioavailability and variable pharmacokinetic behavior [48].

#### 2.4.3. Pharmacokinetic Predictions

In silico ADME analysis predicts that compound **3** has low gastrointestinal absorption and is not blood–brain barrier permeant, consistent with its large molecular size and poor solubility. It shows no major cytochrome P450 interactions and possesses a relatively high predicted skin permeability (log Kp = −3.20 cm/s), aligning with its lipophilic nature.

#### 2.4.4. Drug-likeness and Medicinal Chemistry Filters

**Figure 5:**
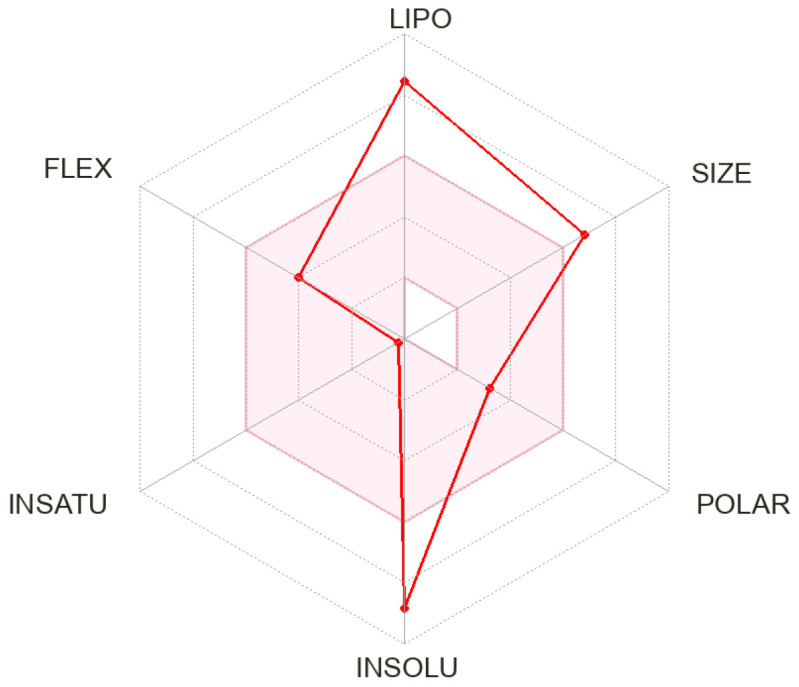
The bioavailability radar for compound **3**.

Compound **3** fails two Lipinski criteria (MW > 500 and MLOGP > 4.15), in addition to violations in the Ghose and Egan filters. Its bioavailability score is 0.17 [49]. A Brenk alert is triggered due to the presence of a peroxide moiety, raising potential concerns about chemical stability and toxicity [50]. These parameters are presented at Figure 5.

Despite its predicted limited aqueous solubility and poor oral bioavailability, compound **3** exhibits a combination of structural and physicochemical properties that may offer distinct therapeutic advantages, particularly in the context of antimalarial drug development. Among its notable features is a high fraction of sp^3^-hybridized carbon atoms (Fsp^3^ = 0.97), which reflects a three-dimensional structure often associated with improved target selectivity, reduced off-target interactions, and enhanced clinical progression [46]. In addition, compound **3** contains eight hydrogen bond acceptors, allowing for increased potential to engage in polar interactions with protein targets, which may enhance binding affinity and specificity [51]. Although highly lipophilic (consensus LogP = 7.54), this characteristic can support passive diffusion across lipid-rich membranes [52]. Furthermore, compound **3** demonstrates a relatively favorable predicted skin permeability (Log Kp = −3.20 cm/s), suggesting its potential use in topical or transdermal applications [53]. The trifluoromethyl substituent contributes to increased metabolic stability, modulates the electronic environment, and may promote halogen bonding with enzyme active sites commonly leveraged in modern drug design [54,55].

### 2.5. Predicted Biological Activities of Compound **3**

In silico prediction using the computer program PASS (v2.0) (Prediction of Activity Spectra for Substances) (https://way2drug.com) has been successfully applied to natural product scaffolds. Using PASS, some additional biological activities could be predicted, which point toward new possible applications of these compounds. This well-established approach serves as a valuable tool for the rational design and selection of promising biological active compounds. For example, PASS prediction for natural polycyclic endoperoxides has found that ~65% of them showed *Pa* values between 0.70 and 0.90 for antiprotozoal activity [56]. According to PASS, plant hydroperoxides demonstrated a wide range of biological activities with antineoplastic and anti-ulcerative as the most expected [57], while for natural steroid and triterpenoid endoperoxy and hydroperoxyl derivatives antihypercholesterolemic, ovulation inhibitory, or anticancer effects are possible with *Pa* > 0.90 [58,59].

PASS analysis has been used for the new hybrid molecule **3** with a bile acid core and spiro-1,2,4-trioxolane moiety. The results of predicted bioactivities are shown in Table 3 with *Pa* (probability of activity) > 0.5 [60]. As one can see, compound **3** could possess a broad spectrum of activities with *Pa* values varying between 0.908 and 0.501 such as antiprotozoal (active against several parasitic infections, such as malaria, amebiasis, giardiasis, cryptosporidiosis, trypanosomiasis leishmaniasis, and toxoplasmosis), anti-inflammatory and dermatological effects (antieczematic, antipruritic), influence on lipid metabolism (enzyme inhibition, cholesterol antagonism), and potential anticancer or chemopreventive activity (adenomatous polyposis treatment). These predictions are consistent with those previously reported for natural and synthetic steroid and terpene peroxides, which are well-known for strong antiparasitic effects and modulation of lipid-related pathways [14]. Moderate probabilities were also observed for dermatological effects and antiviral activity against rhinovirus which align with emerging reports of membrane-interacting endoperoxides exhibiting moderate inhibition of RNA viruses [61].

Collectively, the obtained in silico data for compound **3** within the established profile of peroxide-based bioactive molecules reveal the potency of this steroidal hybrid for further exploration.

## 3. Materials and Methods

The ^1^H and ^13^C NMR spectra were recorded on a “Bruker Avance-III” (Bruker, Billerica, MA, USA, 500 and 125.5 MHz, respectively, δ, ppm, Hz) in CDCl_3_, internal stand-ardtetramethylsilane. Mass spectra were obtained on a liquid high-resolution chromatograph–mass spectrometer Agilent LC/Q-TOF 6530 (Agilent Technologies, Santa Clara, CA, USA). Melting points were detected on a microtable «Rapido PHMK05» (Nagema, Dresden, Germany). Optical rotations were measured on a polarimeter Perkin-Elmer 241 MC (PerkinElmer, Waltham, MA, USA) in a tube length of 1 dm. Elemental analysis was performed on a Euro EA-3000 CHNS analyzer (Eurovector, Milan, Italy); the main standard is acetanilide. Thin-layer chromatography analyses were performed on Sorbfil plates (Sorbpolimer, Krasnodar, Russia), using the solvent system chloroform–ethyl acetate, 40:1. Substances were detected by a 10% solution of sulfuric acid solution with subsequent heating at 100–120 °C for 2–3 min. All chemicals were of reagent grade (Sigma-Aldrich, St. Louis, MO, USA). XRD data for compound **3** were obtained on a Bruker Kappa Apex II CCD diffractometer using φ, ω scans of narrow (0.8°) frames with Mo Kα radiation (λ = 0.71073 Å) and a graphite monochromator at T = 296(2) K. The structure was solved by direct methods using the SHELXT-2014/5 [62] and refined by full-matrix least-squares method against all F2 in anisotropic approximation using the SHELXL-2018/3 [62]. Absorption corrections were applied using the empirical multi-scan method with the SADABS program (Version 2008/1) [63]. Compound **1** was obtained according to the method described previously [35].

### 3.1. The Procedure for Synthesis of Compound **2**

CH_3_ONH_2_·HCl (0.17 g, 2 mmol) was added to the solution of compound **1** (0.39 g, 1 mmol) in a mixture of pyridine and methanol (30 mL, 1:1, *v*/*v*). The reaction mixture was refluxed for 8 h with a back condenser, cooled to room temperature, and quenched with 5% HCl (150 mL). The precipitate was filtered off, washed with water, and airdried. The yield of amorphous substance was 0.39 g (93%) (compounds **2a** and **2b**), [α]^20^_D_ +14 (c 0.10, CH_2_Cl_2_).

#### 3.1.1. Methyl-O-methyl-3(Z)-oxyimino-5β-cholan-24-oate (**2a**)

^1^H NMR (CDCl_3_, δ ppm, *J* Hz): 0.64 (s, 3H, H-18); 0.89 (d, 3H, ^3^*J*_21-20_ = 6.5, H-21); 0.94 (s, 3H, H-19); 1.04 (m, 1H, H-14); 1.05 (m, 1H, H_β_-15); 1.07 (m, 1H, H_ax_-7); 1.08 (m, 1H, H-17); 1.14 (m, 1H, H_ax_-1); 1.15 (m, 1H, H_ax_-12); 1.26 (m, 1H, H_β_-16); 1.26 (m, 1H, H_ax_-11); 1.28 (m, 1H, H_eq_-6); 1.30 (m, 1H, H_A_-22); 1.35 (m, 1H, H-9); 1.38 (m, 1H, H-20); 1.39 (m, 1H, H-8); 1.41 (m, 1H, H_eq_-11); 1.44 (m, 1H, H_eq_-7); 1.48 (m, 1H, H-5); 1.56 (m, 1H, H_α_-15); 1.77 (m, 1H, H_B_-22); 1.83 (m, 1H, H_α_-16); 1.84 (m, 1H, H_ax_-6); 1.87 (m, 1H, H_eq_-1); 1.96 (m, 1H, H_eq_-12); 2.07 (td, 1H, ^2^*J* = 14.8, ^3^*J*_2ax-1ax_ = 14.8, ^3^*J*_2ax-1eq_ = 4.8, H_ax_-2); 2.11 (dd, 1H, ^2^*J* = 15.5, ^3^*J*_4ax-5_ = 13.4, H_ax_-4); 2.12 (m, 1H, H_eq_-2); 2.19 (ddd, 1H, ^2^*J* = 15.4, ^3^*J*_23A-22A_ = 9.7, ^3^*J*_23A-22B_ = 6.5, H_A_-23); 2.33 (ddd, 1H, ^2^*J* = 15.4, ^3^*J*_23B-22B_ = 10.1, ^3^*J*_23B-22A_ = 5.2, H_B_-23); 2.79 (ddd, 1H, ^2^*J* = 15.5, ^3^*J*_4eq-5_ = 5.0, ^4^*J*_4eq-2eq_ = 1.5, H_eq_-4); 3.64 (s, 3H, OMe); 3.79 (s, 3H, NOCH_3_). ^13^C NMR (CDCl_3_, δ ppm): 12.05 (C18); 18.26 (C21); 21.05 (C11); 23.13 (C19); 24.16 (C15); 25.69 (C4); 25.92 (C7); 26.61 (C6); 26.90 (C2); 28.15 (C16); 30.98 (C22); 31.02 (C23); 35.28 (C10); 35.35 (C20); 35.58 (C8); 37.11 (C1); 40.06 (C12); 40.55 (C9); 42.50 (C5); 42.74 (C13); 51.46 (OMe); 55.94 (C17); 56.44 (C14); 60.97 (NOCH_3_); 160.94 (C3); 174.71 (C24). ^15^N NMR (CDCl_3_, δ ppm): 357.33 (NOCH_3_). Anal. Calcd. for C_26_H_43_NO_3_: C, 74.78 H, 10.38; N, 3.35. Found: C, 74.56; H, 10.26; N, 3.11.

#### 3.1.2. Methyl-O-methyl-3(E)-oxyimino-5β-cholan-24-oate (**2b**)

^1^H NMR (CDCl_3_, δ ppm, *J* Hz): 0.64 (s, 3H, H-18); 0.89 (d, 3H, ^3^*J*_21-20_ = 6.5, H-21); 0.93 (s, 3H, H-19); 1.04 (m, 1H, H-14); 1.05 (m, 1H, H_β_-15); 1.06 (m, 1H, H_ax_-1); 1.07 (m, 1H, H_ax_-7); 1.08 (m, 1H, H-17); 1.15 (m, 1H, H_ax_-12); 1.26 (m, 1H, H_β_-16); 1.26 (m, 1H, H_ax_-11); 1.28 (m, 1H, H_eq_-6); 1.30 (m, 1H, H_A_-22); 1.38 (m, 1H, H-20); 1.39 (m, 1H, H-9); 1.39 (m, 1H, H-8); 1.41 (m, 1H, H_eq_-11); 1.44 (m, 1H, H_eq_-7); 1.54 (m, 1H, H-5); 1.56 (m, 1H, H_α_-15); 1.69 (td, 1H, ^2^*J* = 14.6, ^3^*J*_2ax-1ax_ = 14.6, ^3^*J*_2ax-1eq_ = 4.8, H_ax_-2); 1.77 (m, 1H, H_B_-22); 1.83 (m, 1H, H_α_-16); 1.84 (m, 1H, H_eq_-1); 1.84 (m, 1H, H_ax_-6); 1.94 (ddd, 1H, ^2^*J* = 14.6, ^3^*J*_4eq-5_ = 4.8, ^4^*J*_4eq-2eq_ = 2.0, H_eq_-4); 1.96 (m, 1H, H_eq_-12); 2.19 (ddd, 1H, ^2^*J* = 15.4, ^3^*J*_23A-22A_ = 9.7, ^3^*J*_23A-22B_ = 6.5, H_A_-23); 2.33 (ddd, 1H, ^2^*J* = 15.4, ^3^*J*_23B-22B_ = 10.1, ^3^*J*_23B-22A_ = 5.2, H_B_-23); 2.49 (dd, 1H, ^2^*J* = 14.6, ^3^*J*_4ax-5_ = 13.3, H_ax_-4); 2.95 (dddd, 1H, ^2^*J* = 14.6, ^3^*J*_2eq-1ax_ = 4.5, ^3^*J*_2eq-1eq_ = 2.6, ^4^*J*_2eq-4eq_ = 2.0, H_eq_-2); 3.64 (s, 3H, OMe); 3.78 (s, 3H, NOCH_3_). ^13^C NMR (CDCl_3_, δ ppm): 12.05 (C18); 18.27 (C21); 20.48 (C2); 21.05 (C11); 23.02 (C19); 24.16 (C15); 25.98 (C7); 26.61 (C6); 28.15 (C16); 30.98 (C22); 31.02 (C23); 32.20 (C4); 35.35 (C20); 35.36 (C10); 35.61 (C8); 36.17 (C1); 40.10 (C12); 40.33 (C9); 42.74 (C13); 44.07 (C5); 51.46 (OMe); 55.94 (C17); 56.46 (C14); 60.94 (NOCH_3_); 161.09 (C3); 174.71 (C24). ^15^N NMR (CDCl_3_, δ ppm): 355.36 (NOCH_3_). Anal. Calcd. for C_26_H_43_NO_3_: C, 74.78 H, 10.38; N, 3.35. Found: C, 74.56; H, 10.26; N, 3.11.

### 3.2. The Procedure of Griesbaum Co-Ozonolysis

Ozone was bubbled through a solution of compound **2** (0.47 g, 1 mmol) in a mixture of cyclohexane and CH_2_Cl_2_ (30 mL, 1:2, *v*/*v*) in the presence of 4-(trifluoromethyl)-cyclohexanone (0.28 mL, 2 mmol) at 0 °C with TLC control. After completion of the reaction, the solution was flushed with oxygen for 5 min before being concentrated in vacuo at room temperature to give a residue that was then purified by column chromatography (hexane, benzene, and chloroform as the eluents) to afford compounds **3** (0.41g, 72%) and **4** (0.1 g, 24%).

#### 3.2.1. (3S)-3,5′-dispiro-[(4″-trifluoromethyl-cyclohexyl)-1′,2′,4′-trioxolane]-5β-cholan-24-oate (**3**)

Obtained by crystallization from Et_2_O. [α]^20^_D_ + 45° (c 0.10, CHCl_3_). mp. 101 °C. ^1^H NMR (CDCl_3_, δ ppm, *J* Hz): 0.64 (s, 3H, H-18); 0.91 (d, 3H, ^3^*J*_21-20_ = 6.5, H-21); 0.93 (s, 3H, H-19); 1.01 (m, 1H, H-14); 1.03 (m, 1H, H_β_-15); 1.04 (m, 1H, H_ax_-7); 1.08 (m, 1H, H-17); 1.11 (td, 1H, ^2^*J* = 12.5, ^3^*J*_12ax-11ax_ = 12.5, ^3^*J*_12ax-11eq_ = 4.4, H_ax_-12); 1.21 (m, 1H, H_ax_-1); 1.22 (m, 1H, H_eq_-6); 1.25 (m, 1H, H_ax_-11); 1.28 (m, 1H, H_β_-16); 1.31 (m, 1H, H-9); 1.33 (m, 1H, H_A_-22); 1.35 (m, 1H, H_eq_-11); 1.37 (m, 1H, H-8); 1.42 (m, 1H, H-20); 1.42 (m, 1H, H_eq_-7); 1.51 (ddd, 1H, ^2^*J* = 13.6, ^3^*J*_4eq-5_ = 4.2, ^4^*J*_4eq-2eq_ =1.4, H_eq_-4); 1.55 (m, 1H, H-5); 1.56 (m, 1H, H_α_-15); 1.60 (m, 1H, H_ax_-5″); 1.61 (m, 1H, H_ax_-3″); 1.64 (m, 1H, H_ax_-6″); 1.67 (m, 1H, H_ax_-2″); 1.68 (m, 2H, H-2); 1.71 (dt, 1H, ^2^*J* = 11.3, ^3^*J*_1eq-2ax_ = 3.2, ^3^*J*_1eq-2eq_ = 3.2, H_eq_-1); 1.79 (m, 1H, H_B_-22); 1.82 (m, 1H, H_ax_-6); 1.83 (m, 1H, H_α_-16); 1.91 (m, 1H, H_eq_-3″); 1.92 (m, 1H, H_eq_-5″); 1.96 (dt, 1H, ^2^*J* = 12.6, ^3^*J*_12eq-11ax_ = 3.1, ^3^*J*_12eq-11eq_ = 3.1, H_eq_-12); 2.02 (m, 1H, H_eq_-2″); 2.03 (m, 1H, H-4″); 2.05 (m, 1H, H_eq_-6″); 2.15 (t, 1H, ^2^*J* = 13.6, ^3^*J*_4ax-5_ = 13.6, H_ax_-4); 2.22 (ddd, 1H, ^2^*J* = 15.4, ^3^*J*_23A-22A_ = 9.7, ^3^*J*_23A-22B_ = 6.5, H_A_-23); 2.35 (ddd, 1H, ^2^*J* = 15.4, ^3^*J*_23B-22B_ = 10.2, ^3^*J*_23B-22A_ = 5.2, H_B_-23); 3.66 (s, 3H, OMe). ^13^C NMR (CDCl_3_, δ ppm): 12.05 (C18); 18.27 (C21); 21.05 (C11); 22.53 (C3″); 22.58 (C5″); 22.99 (C19); 24.17 (C15); 25.96 (C7); 26.41 (C6); 28.18 (C16); 29.57 (C2); 31.00 (C22); 31.08 (C23); 32.74 (C2″); 32.80 (C6″); 34.02 (C1); 34.32 (C10); 34.79 (C4); 35.37 (C20); 35.55 (C8); 39.74 (C9); 40.14 (C12); 40.42 (q, ^2^*J*_CF_ = 27.3, C4″); 40.82 (C5); 42.75 (C13); 51.49 (OMe); 55.99 (C17); 56.54 (C14); 107.05 (C3′); 110.33 (C3); 127.38 (q, ^1^*J*_CF_ =278.3, CF_3_); 174.77 (C24). ^19^F NMR (CDCl_3_, δ ppm): -73.26 (d, ^2^*J*_FH_ = 8.1, CF_3_). Anal. Calcd. for C_32_H_49_F_3_O_5_: C, 67.34 H, 8.65; F, 9.99. Found: C, 67.19; H, 8.57; F, 9.81.

Crystallographic data for **3**: C_22_H_49_F_3_O_5_, FW = 570.71, Orthorhombic, P2_1_2_1_2_1_, *a* = 6.588(1), *b* = 22.318(4), *c* = 42.214(8) Ǻ, V = 6207(2) Å^3^, Z = 8, Dcalc. = 1.221 g cm^−3^, μ (Mo-Kα) = 0.092 mm^−1^, F(000) = 2464, 25040 measured reflections (θmax = 25.03°, completeness 99.8%), 10933 independent (Rint = 0.080), 721 parameters, R1 = 0.0909 (for 5846 observed I > 2σ(I)), wR2 = 0.1856 (all data), GooF = 1.06, largest diff. peak and hole 0.26 and −0.26 × 10^−3^. Quite high value of the R-factor is explained by bad quality of the crystal; it was not succeeded to receive crystals of the best quality. CCDC 2493448 contains the supplementary crystallographic data for this paper. These data can be obtained from the Cambridge Crystallographic Data Centre via www.ccdc.cam.ac.uk/data_request/cif, accessed on 25 November 2025.

#### 3.2.2. Methyl 4-oxo-3-methoxyaza-A-homo-5β-cholan-24-oate (**4a**)

^1^H NMR (CDCl_3_, δ ppm, *J* Hz): 0.66 (s, 3H, H-18); 0.86 (m, 1H, H_ax_-7); 0.91 (d, 3H, ^3^*J*_21-20_ = 6.5, H-21); 1.00 (s, 3H, H-19); 1.05 (m, 1H, H-14); 1.06 (m, 1H, H_β_-15); 1.11 (dt, 1H, ^3^*J*_17-20_ = 10.1, ^3^*J*_17-16α_ = 9.5, ^3^*J*_17-16β_ = 9.5, H-17); 1.18 (m, 1H, H_ax_-12); 1.22 (m, 1H, H-9); 1.29 (m, 1H, H_β_-16); 1.33 (m, 1H, H_eq_-11); 1.33 (m, 1H, H_A_-22); 1.34 (m, 1H, H_ax_-11); 1.34 (m, 1H, H-8); 1.43 (m, 1H, H-20); 1.44 (m, 1H, H_eq_-6); 1.46 (m, 1H, H_eq_-7); 1.58 (m, 1H, H_α_-15); 1.61 (dd, 1H, ^2^*J* = 15.4, ^3^*J*_1ax-2ax_ = 9.1, H_ax_-1); 1.79 (m, 1H, H-5); 1.80 (m, 1H, H_B_-22); 1.81 (dd, 1H, ^2^*J* = 15.4, ^3^*J*_1eq-2eq_ = 9.1, H_eq_-1); 1.86 (m, 1H, H_α_-16); 1.87 (m, 1H, H_ax_-6); 1.99 (m, 1H, H_eq_-12); 2.16 (d, 1H, ^2^*J* = 15.3, H_eq_-4a); 2.22 (ddd, 1H, ^2^*J* = 15.4, ^3^*J*_23A-22A_ = 9.7, ^3^*J*_23A-22B_ = 6.5, H_A_-23); 2.35 (ddd, 1H, ^2^*J* = 15.4, ^3^*J*_23B-22B_ = 10.1, ^3^*J*_23B-22A_ = 5.2, H_B_-23); 2.95 (dd, 1H, ^2^*J* = 15.3, ^3^*J*_4aax-5_ = 12.0, H_ax_-4a); 3.54 (d, 1H, ^2^*J* = 14.9, ^3^*J*_2eq-1eq_ = 9.1, H_eq_-2); 3.66 (s, 3H, OMe); 3.67 (d, 1H, ^2^*J* = 14.9, ^3^*J*_2ax-1ax_ = 9.1, H_ax_-2); 3.73 (s, 3H, NOCH_3_). ^13^C NMR (CDCl_3_, δ ppm): 12.04 (C18); 18.26 (C21); 21.28 (C11); 22.28 (C19); 24.11 (C15); 26.16 (C7); 28.10 (C16); 29.58 (C6); 30.96 (C22); 31.05 (C23); 35.34 (C20); 35.71 (C8); 36.35 (C10); 37.83 (C4a); 38.56 (C1); 39.68 (C5); 40.03 (C12); 42.61 (C13); 43.76 (C9); 45.95 (C2); 51.51 (OMe); 55.95 (C17); 56.14 (C14); 61.61 (NOCH_3_); 171.39 (C4); 174.73 (C24). ^15^N NMR (CDCl_3_, δ ppm): 195.68 (NOCH_3_). Anal. Calcd. for C_26_H_43_NO_4_: C, 72.02 H, 10.00; N, 3.23. Found: C, 71.95; H, 9.89; N, 3.01.

#### 3.2.3. Methyl-3-oxo-4-methoxyaza-A-homo-5β-cholan-24-oate (**4b**)

^1^H NMR (CDCl_3_, δ ppm, *J* Hz): 0.66 (s, 3H, H-18); 0.82 (m, 1H, H_ax_-7); 0.91 (d, 3H, ^3^*J*_21-20_ = 6.5, H-21); 0.99 (s, 3H, H-19); 1.06 (m, 1H, H_β_-15); 1.07 (m, 1H, H-14); 1.11 (dt, 1H, ^3^*J*_17-20_ = 10.1, ^3^*J*_17-16α_ = 9.5, ^3^*J*_17-16β_ = 9.5, H-17); 1.18 (m, 1H, H_ax_-12); 1.28 (m, 1H, H-9); 1.29 (m, 1H, H_β_-16); 1.32 (m, 1H, H_eq_-11); 1.33 (m, 1H, H_A_-22); 1.35 (m, 1H, H_ax_-11); 1.37 (m, 1H, H-8); 1.43 (m, 1H, H-20); 1.44 (dd, 1H, ^2^*J* = 15.0, ^3^*J*_1ax-2ax_ = 11.4, H_ax_-1); 1.51 (m, 1H, H_eq_-7); 1.52 (m, 1H, H_eq_-6); 1.58 (m, 1H, H_α_-15); 1.76 (m, 1H, H-5); 1.78 (dd, 1H, ^2^*J* = 15.0, ^3^*J*_1eq-2eq_ = 10.0, H_eq_-1); 1.80 (m, 1H, H_B_-22); 1.86 (m, 1H, H_α_-16); 1.92 (m, 1H, H_ax_-6); 1.99 (m, 1H, H_eq_-12); 2.22 (ddd, 1H, ^2^*J* = 15.4, ^3^*J*_23A-22A_ = 9.7, ^3^*J*_23A-22B_ = 6.5, H_A_-23); 2.32 (d, 1H, ^2^*J* = 15.9, ^3^*J*_2eq-1eq_ = 10.0, H_eq_-2); 2.35 (ddd, 1H, ^2^*J* = 15.4, ^3^*J*_23B-22B_ = 10.1, ^3^*J*_23B-22A_ = 5.2, H_B_-23); 2.50 (d, 1H, ^2^*J* = 15.9, ^3^*J*_2ax-1ax_ = 11.4, H_ax_-2); 3.20 (d, 1H, ^2^*J* = 15.2, H_eq_-4a); 3.66 (s, 3H, OMe); 3.75 (s, 3H, NOCH_3_); 4.25 (dd, 1H, ^2^*J* = 15.2, ^3^*J*_4aax-5_ = 11.0, H_ax_-4a). ^13^C NMR (CDCl_3_, δ ppm): 12.03 (C18); 18.26 (C21); 20.97 (C11); 22.73 (C19); 24.11 (C15); 26.88 (C7); 28.10 (C16); 28.21 (C6); 29.44 (C2); 30.96 (C22); 31.05 (C23); 34.05 (C1); 35.34 (C20); 35.73 (C8); 36.54 (C10); 40.00 (C12); 42.64 (C13); 42.90 (C9); 43.56 (C5); 51.51 (OMe); 53.27 (C4a); 55.95 (C17); 56.21 (C14); 61.43 (NOCH_3_); 171.55 (C4); 174.73 (C24). ^15^N NMR (CDCl_3_, δ ppm): 192.78 (NOCH_3_). Anal. Calcd. for C_26_H_43_NO_4_: C, 72.02 H, 10.00; N, 3.23. Found: C, 71.95; H, 9.89; N, 3.01.

### 3.3. Biological Activity Assays

Viruses and cells: Influenza virus A/Puerto Rico/8/34 (H1N1) was obtained from the collection of viruses of St. Petersburg Pasteur Institute. Before the experiment, virus was propagated in the allantoic cavity of 10- to 12-day-old chicken embryos for 48 h at 36 °C. The infectious titer of the virus was determined in Madin-Darby canine kidney (MDCK) cells (ATCC-CCL-34) grown in 96-well plates in alpha-MEM medium with 10% fetal bovine serum.

#### 3.3.1. Cytotoxicity Assay

MDCK cells were seeded onto 96-well culture plates (104 cells per well) and incubated at 36 °C in 5% CO_2_ until continuous monolayer formation. To assess the toxicity of compounds, a series of their 3-fold dilutions at concentrations of 300 to 3.7 μg/mL in Eagle’s Minimal Essential Medium (MEM) were prepared. The dilutions were added to the wells of the plates. Cells were incubated for 72 h at 36 °C in a CO2 incubator under 5% CO2. Further, a microtetrazolium (MTT) assay was performed on 96-well plates. The cells were washed 2 times with saline (0.9% NaCl), and 100 μL/well of MTT solution [3-(4,5-dimethylthiazol-2-yl)-2,5-diphenyltetrazolium bromide] at a concentration of 0.5 μg/mL in MEM was added. The plates were incubated for 1 h at 36 °C, the liquid was removed, and dimethylsulfoxide (DMSO) (0.1 mL per well) was added. The optical density (OD) of the cells was measured on a Thermo Multiskan FC spectrophotometer (Thermo Fisher Scientific, Waltham, MA, USA) at a wavelength of 540 nm. Based on the obtained data, the CC_50_, the concentration of the compound that destroys 50% of the cells in the culture, was calculated for each specimen.

#### 3.3.2. CPE Reduction Assay

The compounds in appropriate concentrations were added to MDCK cells (0.1 mL per well). MDCK cells were further infected with A/Puerto Rico/8/34 (H1N1) influenza virus (m.o.i 0.01). Plates were incubated for 72 h at 36 °C at 5% CO_2_. After that, cell viability was assessed by the MTT test, as described above. The cytoprotective activity of compounds was considered as their ability to increase the values of the OD compared to the control wells (with virus only; no drugs). Based on the obtained results, the IC_50_ values, i.e., the concentration of compounds that results in 50% cell protection, were calculated using GraphPad Prism 6.01 software. IC_50_ values in μg/mL were then calculated into micromoles. For each compound, the value of the selectivity index (SI) was calculated as a ratio of CC_50_ to IC_50_.

#### 3.3.3. Statistical Data

Calculations of the 50% cytotoxicity response (CC_50_) and 50% treatment efficacy (IC_50_) results were performed using the GraphPad Prism v.6.0 software package. A four-parameter logistic curve equation was used as the initial model for the analysis (menu items “Nonlinear Regression”–“Logarithm of Inhibitor—Response”). Based on the obtained data, the selectivity index (SI)—the ratio of CC_50_ to IC_50_—is calculated for each compound and each virus. A combination of states is considered active if its selectivity index is 10 or higher [64].

### 3.4. SwissADME

The physicochemical properties of compound **3** were calculated using online software SwissADME (Version 2017/1) (https://www.swissadme.ch/index.php) (accessed on 7 October 2025).

### 3.5. PASS Analysis

PASS (v2.0) (Prediction of Activity Spectra for Substances) analysis was calculated on https://way2drug.com. The output file represents a list of activities with two probabilities *Pa* (probability to be active) and *Pi* (probability to be inactive). *Pa* (probability “to be active”) estimates the chance that the studied compound belongs to the sub-class of active compounds (resembles the structures of molecules, which are the most typical in a sub-set of “actives” in PASS training set). *Pi* (probability “to be inactive”) estimates the chance that the studied compound belongs to the sub-class of inactive compounds (resembles the structures of molecules, which are the most typical in a sub-set of “inactives” in PASS training set). The *Pa* value based on a cut-off of >0.5 [60].

## 4. Conclusions

By the application of a synthetic method of Griesbaum co-ozonolysis, a novel steroid peroxide, namely methyl 3(*S*)-3,5′-dispiro-[(4″-trifluoromethyl-cyclohexyl)-1′,2′,4′-trioxolane]-5*β*-cholan-24-oate, was stereospecifically formed and its structure was established by 2D NMR and X-ray crystallographic analysis. The evaluation of its cytotoxicity and anti-influenza activity against A/Puerto Rico/8/34 (H1N1) showed that steroid 1,2,4-trioxolane **3** exhibited the highest potency (IC_50_ 4.3 µM, SI 11) compared to the parent methyl 3-oxo-litocholate **1** (IC_50_ > 84 µM, SI 1). Despite predicted low aqueous solubility and poor oral bioavailability, compound **3** exhibits several advantageous features such as a three-dimensional structure (Fsp^3^ = 0.97), high lipophilicity (LogP = 7.54), and the presence of key pharmacophores (CF_3_, peroxide, H-bond acceptors) that makes it a promising candidate for further studies. Moreover, introducing polar substituents at the carboxyl group in the side chain (for example, Mannich bases) or other modifications of the parent scaffold of LCA, together with the 1,2,4-trioxolane unit at the C3 position, could generate the next series of bioactive molecules. PASS analysis with *Pa* of >0.5 indicated antiprotozoal, anti-inflammatory, dermatological, and anticancer activities and an influence on lipid metabolism as possible for compound **3**.

## Figures and Tables

**Table 1:** Anti-influenza virus A/PuertoRico/8/34 (H1N1) activity of compounds **1**–**3**.

Compound	CC_50_, μM ^a^	IC_50_, μM ^b^	SI ^c^
**1**	107.4 ± 2.1	84 *	1
**2**	17.0 ± 1.3	7.8 **	2
**3**	47.5 ± 2.2	4.3 ± 0.6	11
Rimantadine	62 ± 4	11 ± 2	6
Oseltamivir	>200	0.3 ± 0.01	>667

^a^ CC_50_ (cytotoxic concentration)—represents the concentration of the compound that causes a 50% reduction in viable host cells; ^b^ IC_50_ (inhibitory concentration)—concentration required to inhibit 50% of viral replication; ^c^ SI—selectivity index is defined as SI CC_50_/IC_50_. * IC_50_ could not be precisely determined; no 50% inhibition observed up to 84 μM. ** IC_50_ could not be precisely determined; no 50% inhibition observed up to 7.8 μM.

**Table 2:** The summary of ADME properties for compound **3** as detected by Swiss ADME.

Compound	3
Physicochemical Properties	
Formula	C_32_H_49_F_3_O_5_
Molecular weight	570.72 g/mol
Num. heavy atoms	40
Num. arom. heavy atoms	0
Fraction Csp3	0.97
Num. rotatable bonds	6
Num. H-bond acceptors	8
Num. H-bond donors	0
Molar refractivity	147.54
TPSA	53.99 Å^2^
Lipophilicity	
Log *P*_o/w_ (iLOGP)	5.65
Log *P*_o/w_ (XLOGP3)	9.27
Log *P*_o/w_ (WLOGP)	9.62
Log *P*_o/w_ (MLOGP)	6.53
Log *P*_o/w_ (SILICOS-IT)	6.66
Consensus log *P*_o/w_	7.54
Water Solubility	
Log *S* (ESOL)	5.65
Solubility	9.27
Class	9.62
Log *S* (Ali)	6.53
Solubility	6.66
Class	7.54
Log *S* (SILICOS-IT)	−8.82
Solubility	8.59 × 10^−7^ mg/mL; 1.50 × 10^−9^ mol/L
Class	Poorly soluble
Pharmacokinetics	
GI absorption	Low
BBB permeant	No
P-gp substrate	No
CYP1A2 inhibitor	No
CYP2C19 inhibitor	Yes
CYP2C9 inhibitor	No
CYP2D6 inhibitor	No
CYP3A4 inhibitor	No
Log *K*_p_ (skin permeation)	−3.20 cm/s
Drug-likeness	
Lipinski	No; 2 violations: MW > 500, MLOGP > 4.15
Ghose	No; 4 violations: MW > 480, WLOGP > 5.6, MR > 130; atoms > 70
Veber	Yes
Egan	No; 1 violation: WLOGP > 5.88
Muegge	No; 1 violation: XLOGP3 > 5
Bioavailability score	0.17
Medicinal Chemistry	
PAINS	0 alert
Brenk	1 alert: peroxide
Leadlikeness	No; 2 violations: MW > 350, XLOGP3 > 3.5
Synthetic accessibility	7.77

**Table 3:** The findings from the QSAR models regarding the prediction of bioactivity for compound **3**.

Compound	Predicted Biological Activities, (*Pa*) *
**3**	Acylcarnitine hydrolase (0.908); Antiprotozoal (0.869); Alkylacetylglycerophosphatase (0.850); Alkenylglycerophosphocholine hydrolase (0.850); Cholesterol antagonism (0.839); Glyceryl-ether monooxygenase (0.785) Antieczematic (0.788); Adenomatous polyposis treatment (0.767); Antipruritic (0.723); Dermatologic (0.689); CYP7 inhibitor (0.597); Antiviral (Rhinovirus) (0.501)

* Only activities with *Pa* (probability of activity) > 0.5 are shown.

## Data Availability

The original contributions presented in this study are included in the article/Supplementary Materials. Further inquiries can be directed to the corresponding author.

## Supplementary Materials

The following supporting information can be down-loaded at https://www.mdpi.com/article/10.3390/molecules30234613/s1, Figures S1–S22 and S24–S33: ^1^H and ^13^C NMR data of compounds **2**–**4**. Figure S23: The structure of compound **3a**. Figure S34: The Bioavailability Radar (**a**) for compound **1**, (**b**) for compound **3**. Table S1: Crystal data and structure refinement for compound **3**. Table S2: The summary of ADME properties for compounds **1** and **3** as detected by Swiss ADME. Ref. [65] is cited in Supplementary Materials.
