# Supplementary material for: A Stereospecific Synthesis and In Vitro Anti-Influenza H1N1 Properties of Lithocholic Acid-Based Spiro-1,2,4-trioxolane
Source: Molecules. 2025 Dec 1;30(23):4613. doi: 10.3390/molecules30234613 (PMC12693687; doi:10.3390/molecules30234613)
Supplement: Supplementary file 1 [file molecules-30-04613-s001.zip › molecules-3982937-supplementary.pdf]

Supplementary Material

A Stereospecific Synthesis and In Vitro Anti-Influenza H1N1 Properties of Lithocholic Acid-Based *Spiro*-1,2,4-trioxolane

Irina Smirnova [1], Alexander Lobov [1], Liana Zakirova [1], Dmitriy Polovyanenko [2], Irina Bagryanskaya [2], Vladimir Zarubaev [3] and Oxana Kazakova [1,*]

[1] Ufa Institute of Chemistry, Ufa Federal Research Centre, Russian Academy of Sciences, 71, pr. Oktyabrya, 450054 Ufa, Russia; si8081@yandex.ru (I.S.); lobovan@anrb.ru (A.L.); liana.zakirova@gmail.com (L.Z.)

[2] N. N. Vorozhtsov Novosibirsk Institute of Organic Chemistry, Siberian Branch of Russian Academy of Sciences (SB RAS), 630090 Novosibirsk, Russia; dpolo@nioch.nsc.ru (D.P.); bagryan@nioch.nsc.ru (I.B.)

[3] Experimental Virology Laboratory, Department of Virology, St. Petersburg Pasteur Institute of Epidemiology and Microbiology, 14 Mira St., 197001 St. Petersburg, Russia; zarubaev@gmail.com

* Correspondence: obf@anrb.ru

Abstract

Bile acids provide a versatile platform for the design of biologically active compounds due to their amphiphilic structure, biocompatibility, and capacity for diverse chemical modifications. Among them lithocholic acid is a promising scaffold for design and re-vealing of new antiviral agents. A novel lithocholic acid-based 3-spiro-1,2,4-trioxolane was synthesized by Griesbaum co-ozonolysis of methyl 3-O-methyl-oximino-lithocholate and 4-(trifluoromethyl)-cyclohexanone, and its structure was confirmed by 2D NMR and X-ray crystallographic analysis. Lithocholic acid derivatives were evaluated for cytotoxicity and anti-influenza activity against A/Puerto Rico/8/34 (H1N1), showing that steroid 1,2,4-trioxolane 3 exhibited the highest potency (IC50 4.3 µM, SI 11) compared to the parent methyl-3-oxo-lithocholate 1 (IC50 > 84 µM, SI 1). In silico ADME predictions revealed several favorable drug-like properties, including a highly three-dimensional structure (Fsp^3 = 0.97), significant lipophilicity (LogP = 7.54), and the presence of key pharmacophores such as a peroxide moiety and a trifluoromethyl group. Taking together, a stereospecific synthesis of a lithocholic acid 3-spiro-1,2,4-trioxolane by Griesbaum co-ozonolysis was realized and a first evidence of anti-influenza activity in the steroid-1,2,4-trioxolane series was found.

Keywords: bile acids; lithocholic acid; 1,2,4-trioxolane; Griesbaum co-ozonolysis; X-ray analysis; ADME; PASS prediction; antiviral.

NMR data

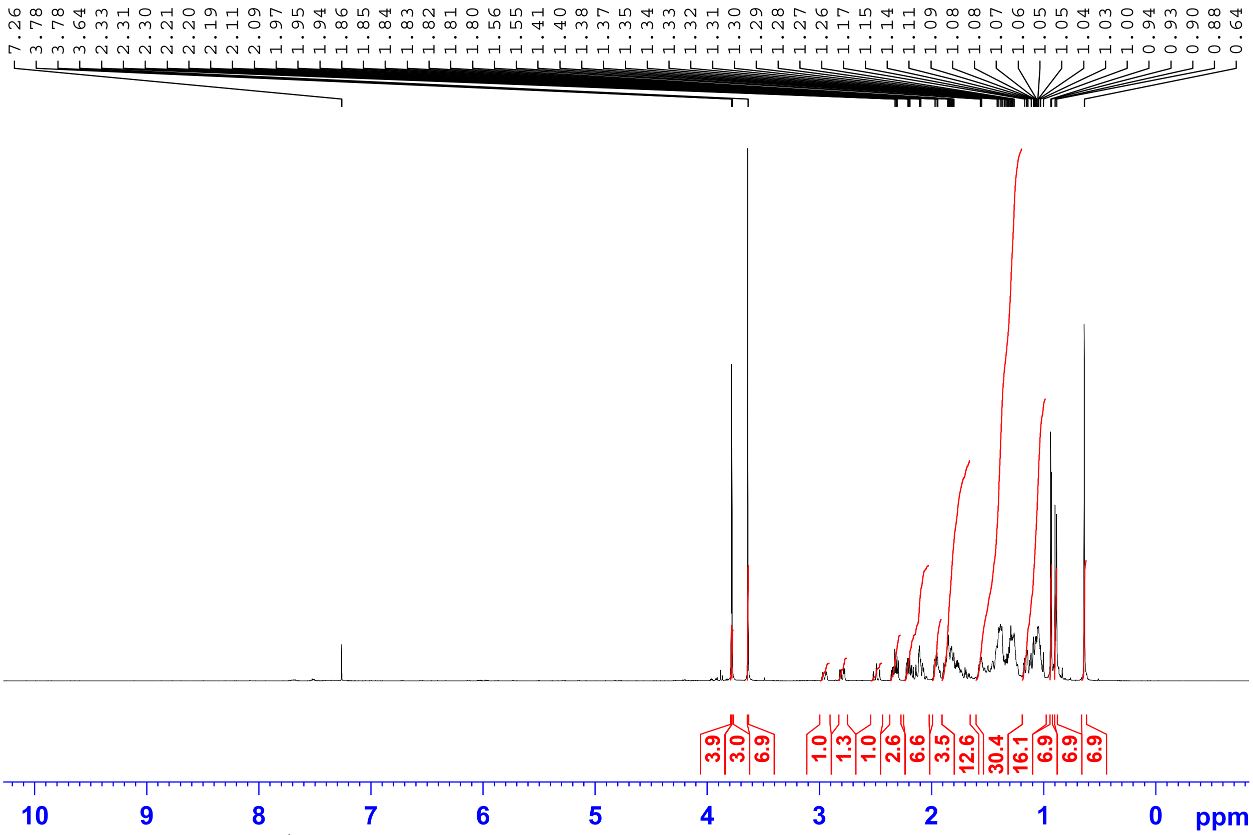

Figure S1. Complete ¹H NMR spectrum of compounds **2a,b** in CDCl₃, 500MHz.

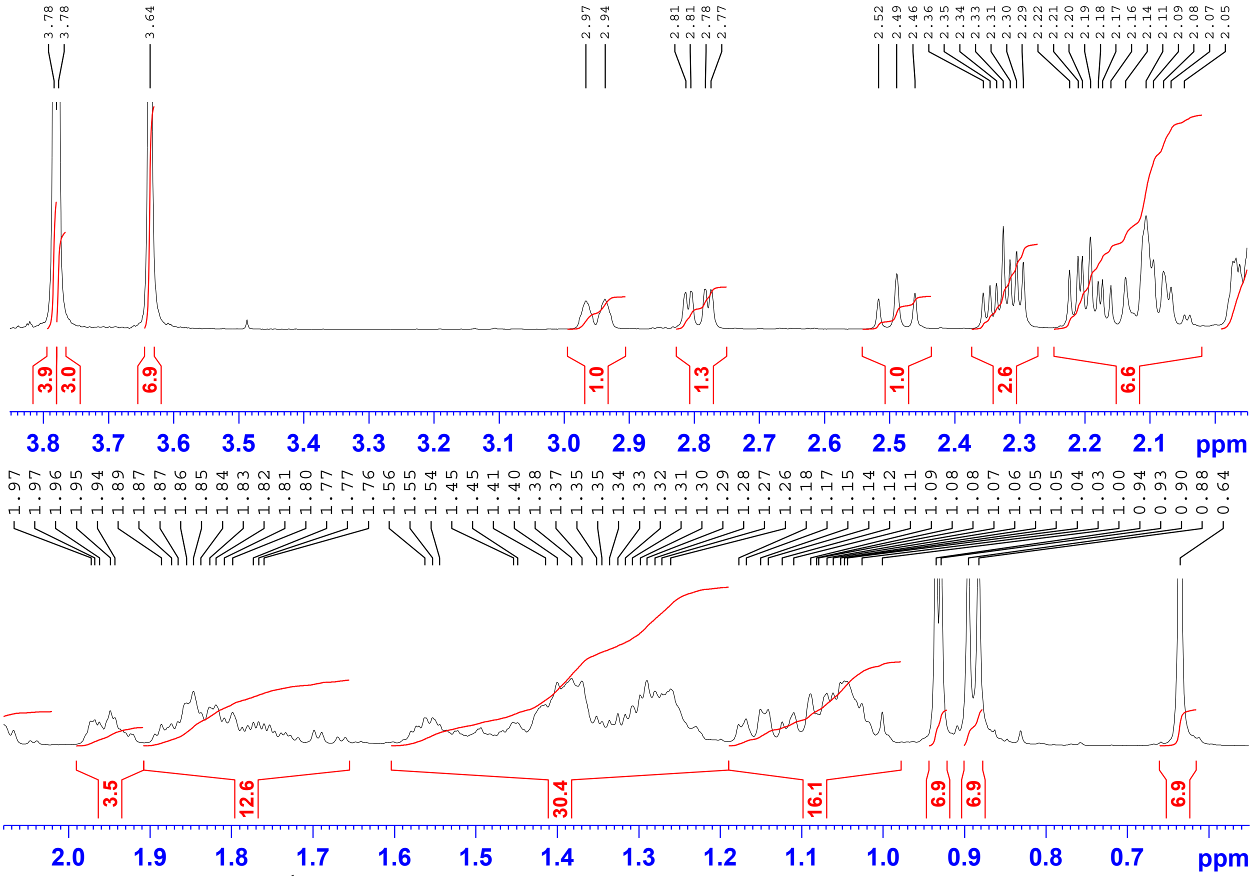

Figure S2. Expanded ¹H NMR spectrum of compounds **2a,b** in CDCl₃, 500MHz.

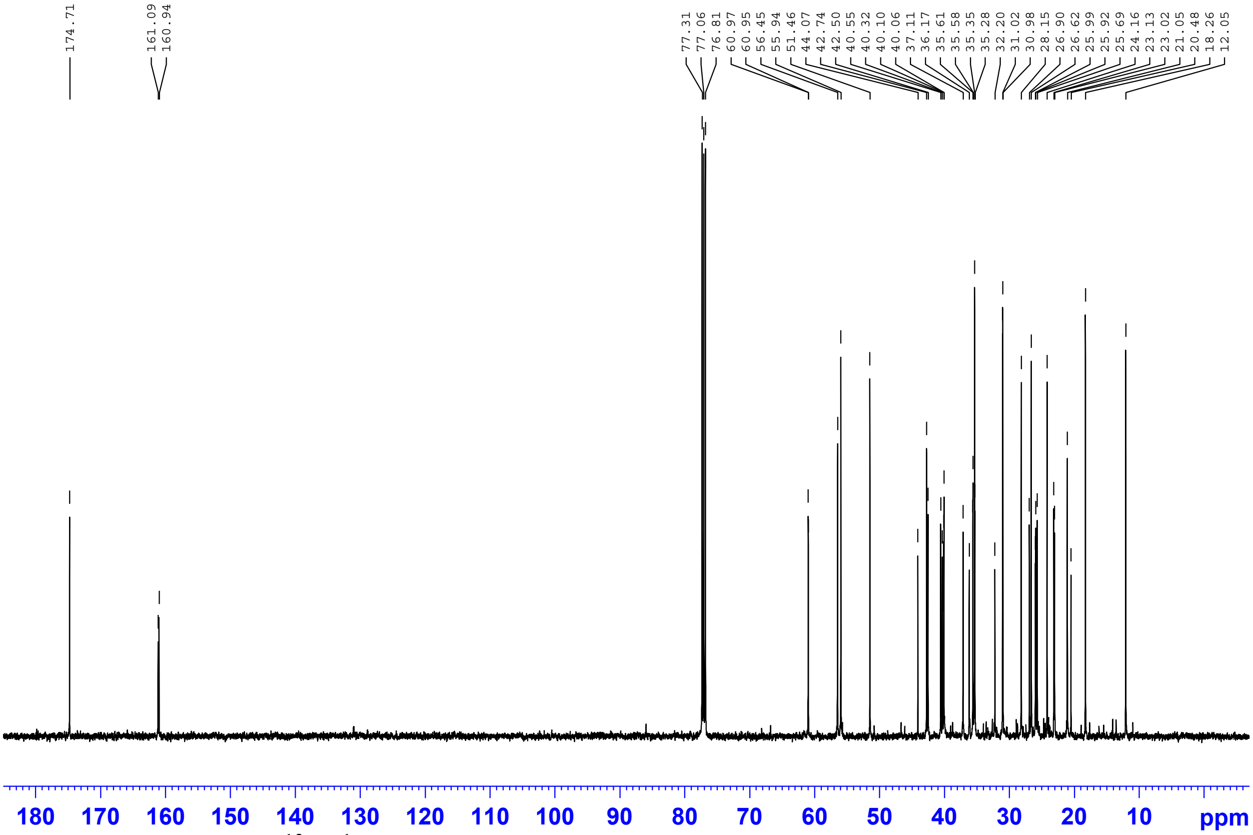

Figure S3. Complete $^{13}C\{^{1}H\}$ spectrum of compounds **2a,b** in $CDCl_3$, 125 MHz.

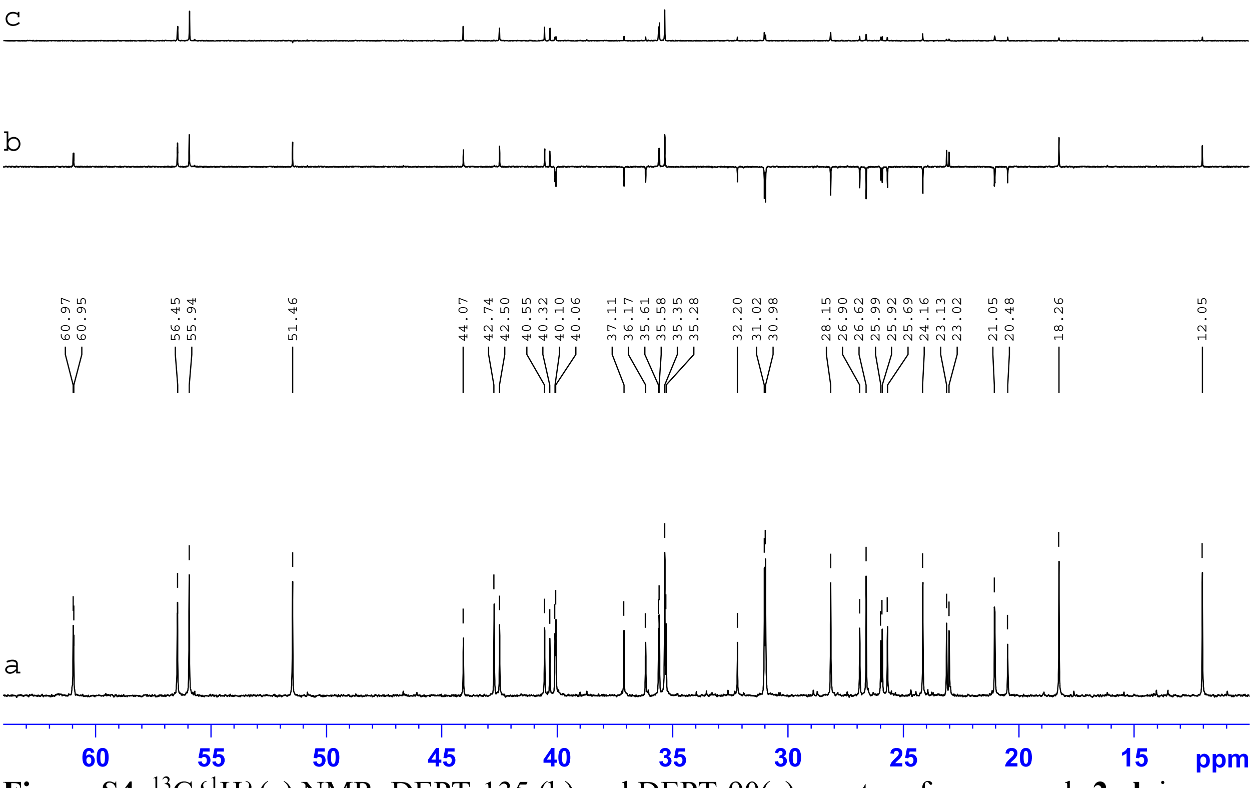

Figure S4. $^{13}C\{^{1}H\}$(a) NMR, DEPT-135 (b) and DEPT-90(c) spectra of compounds **2a,b** in $CDCl_3$, 125 MHz.

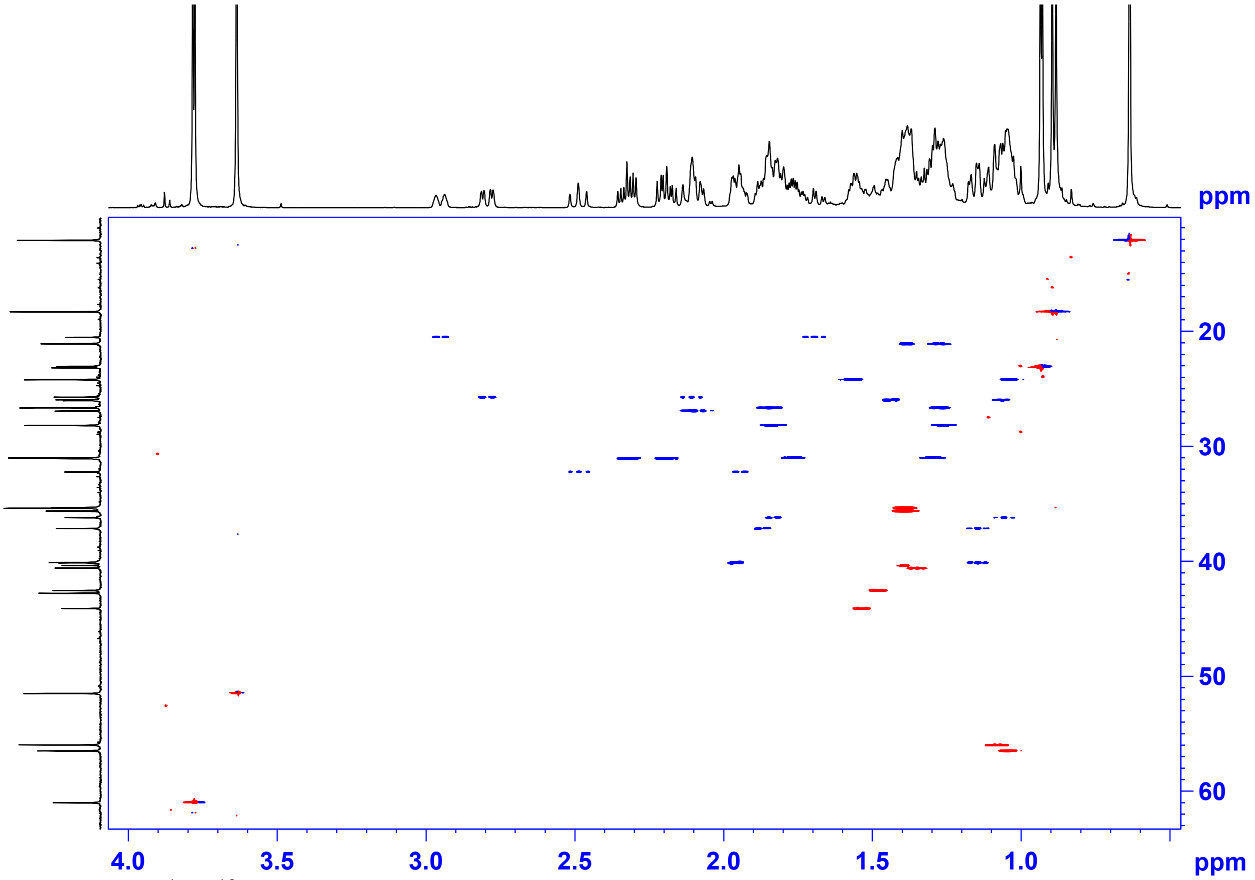

Figure S5. {^{1}H, ^{13}C} HSQCed spectrum of compounds **2a,b** in $CDCl_3$, 500 MHz.

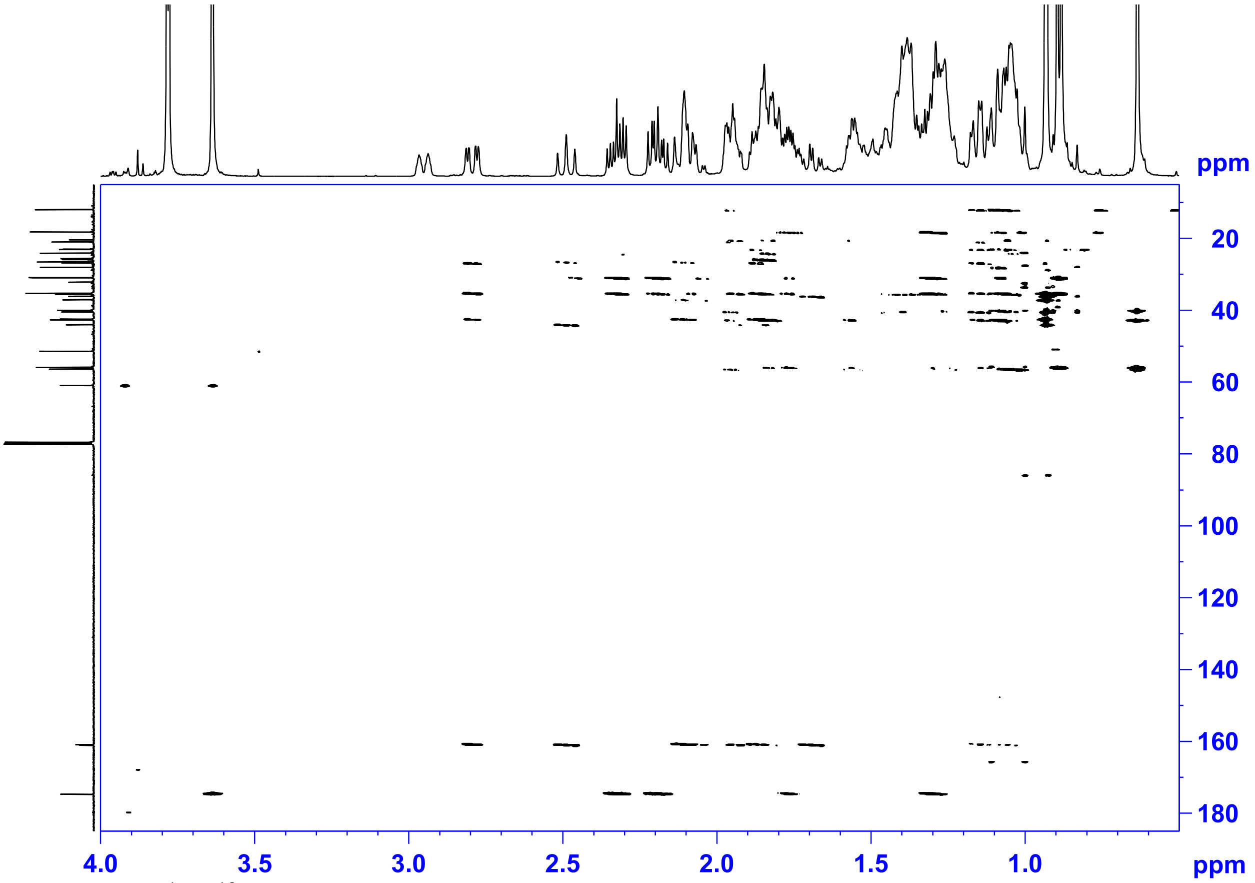

Figure S6. {^{1}H, ^{13}C} HMBC spectrum of compounds **2a,b** in $CDCl_3$, 500 MHz.

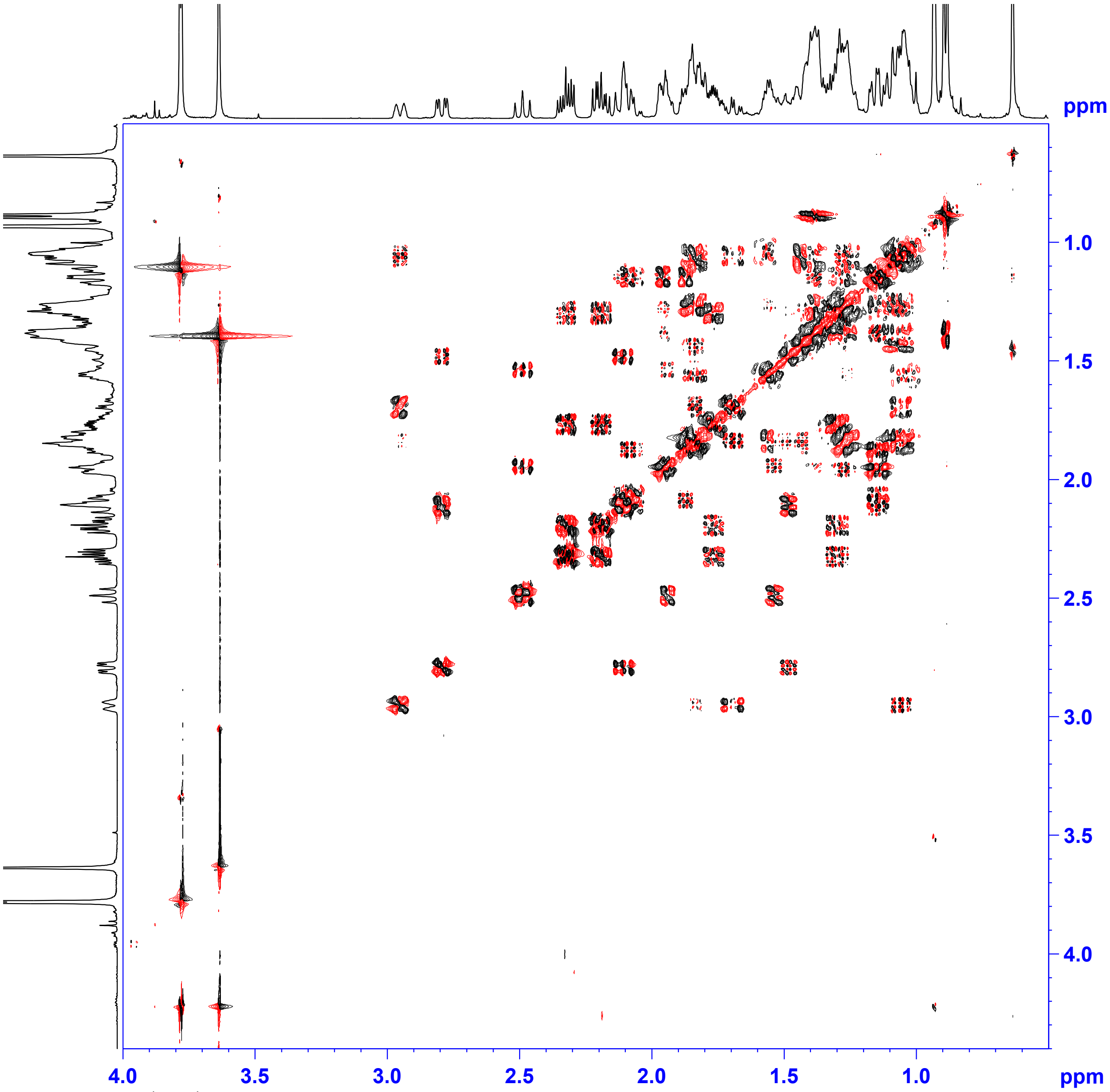

Figure S7. {^{1}H, ^{1}H} COSY-DQF spectrum of compounds **2a,b** in $CDCl_3$, 500 MHz.

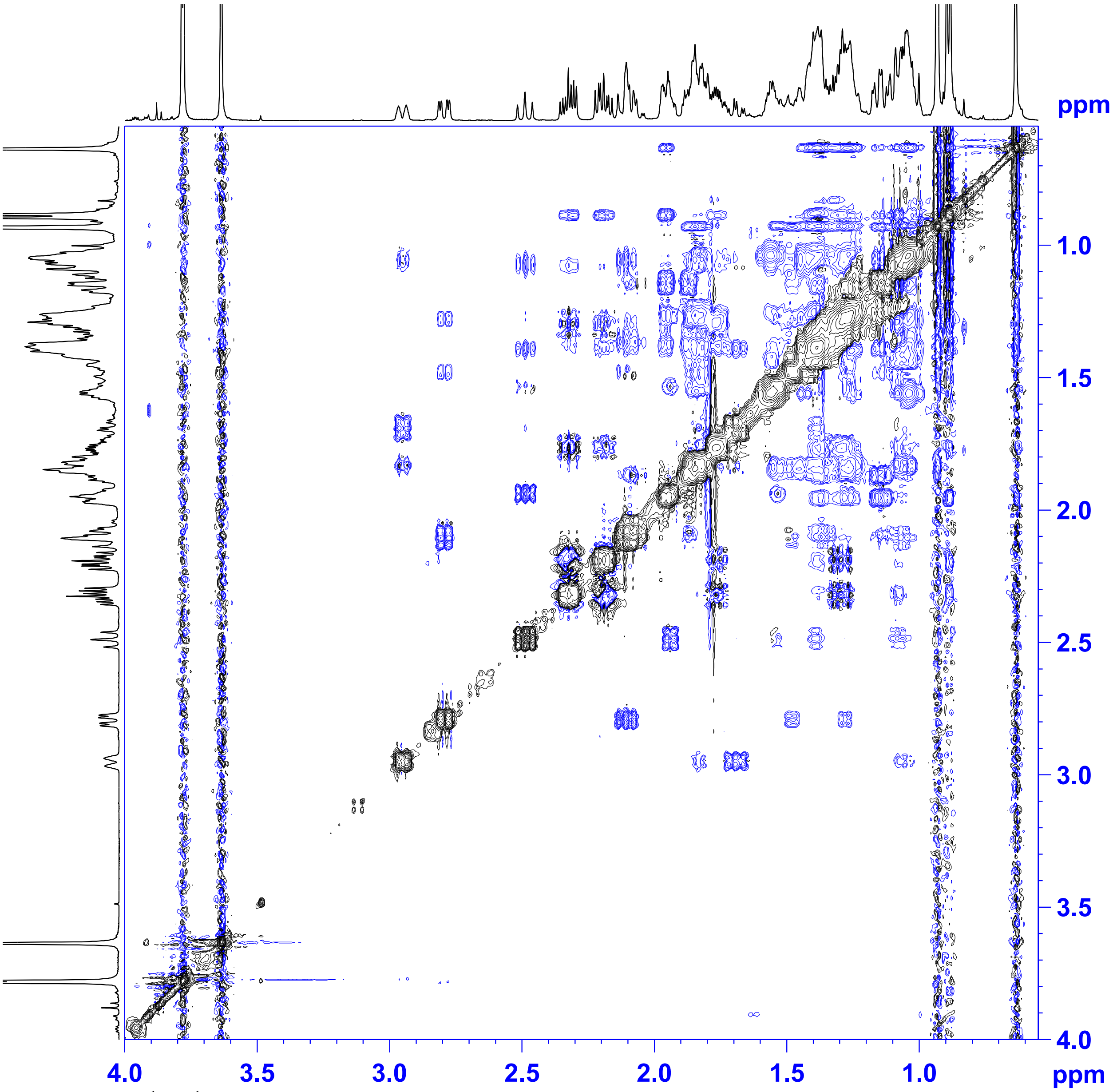

Figure S8. {^{1}H, ^{1}H} NOESY spectrum of compound **2a,b** in $CDCl_3$, 500 MHz.

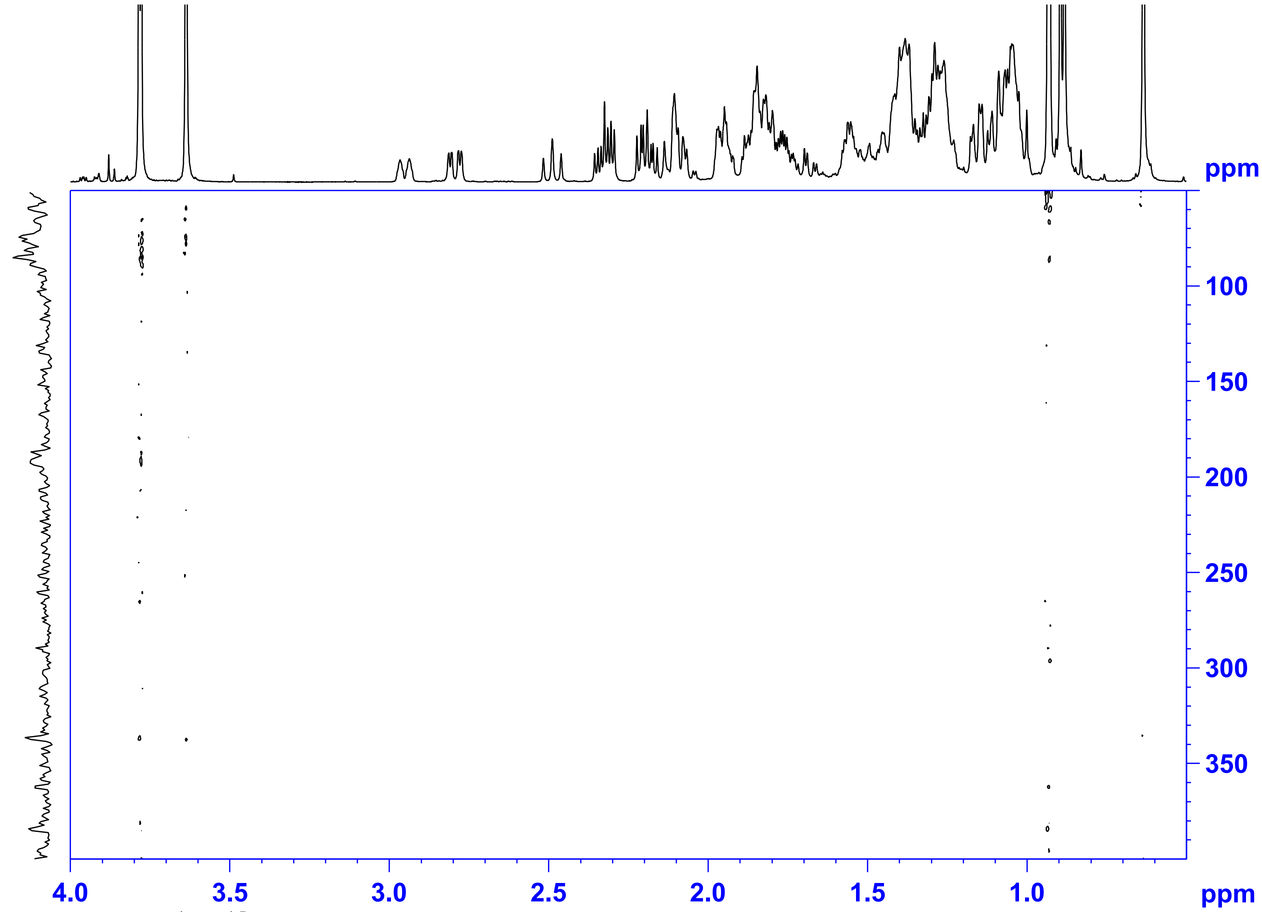

Figure S9. {^{1}H, ^{15}N} HSQC spectrum of compounds **2a,b** in $CDCl_3$, 500 MHz.

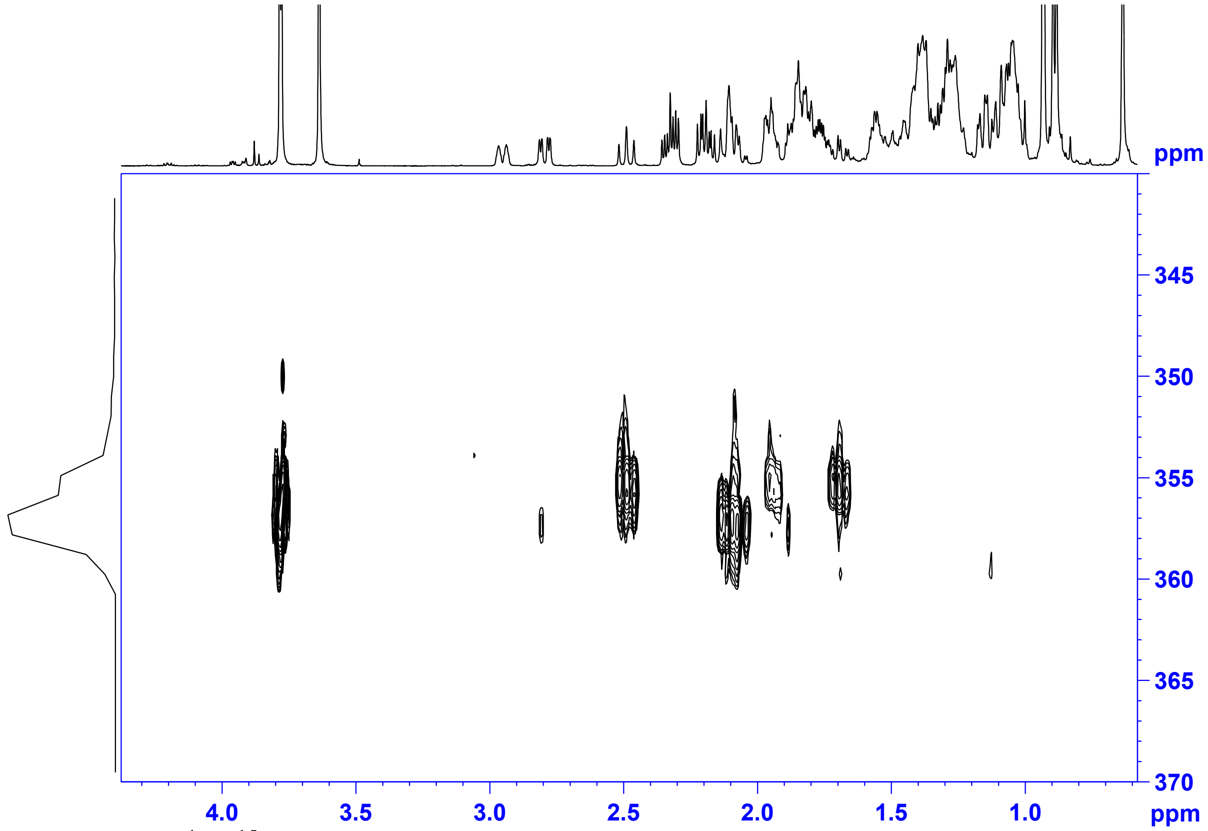

Figure S10. {^{1}H, ^{15}N} HMBC spectrum of compounds **2a,b** in $CDCl_3$, 500 MHz.

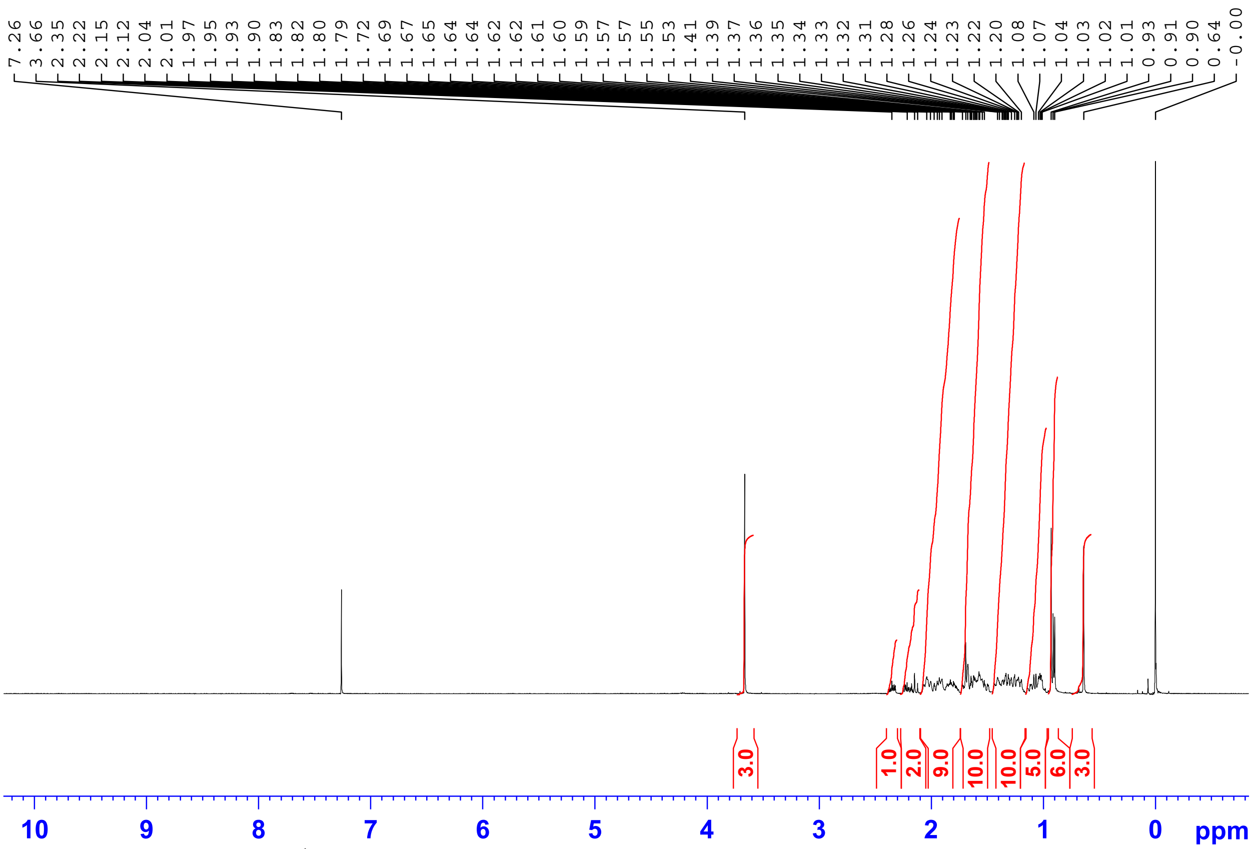

Figure S11. Complete ^{1}H NMR spectrum of compound **3** in $CDCl_3$, 500MHz.

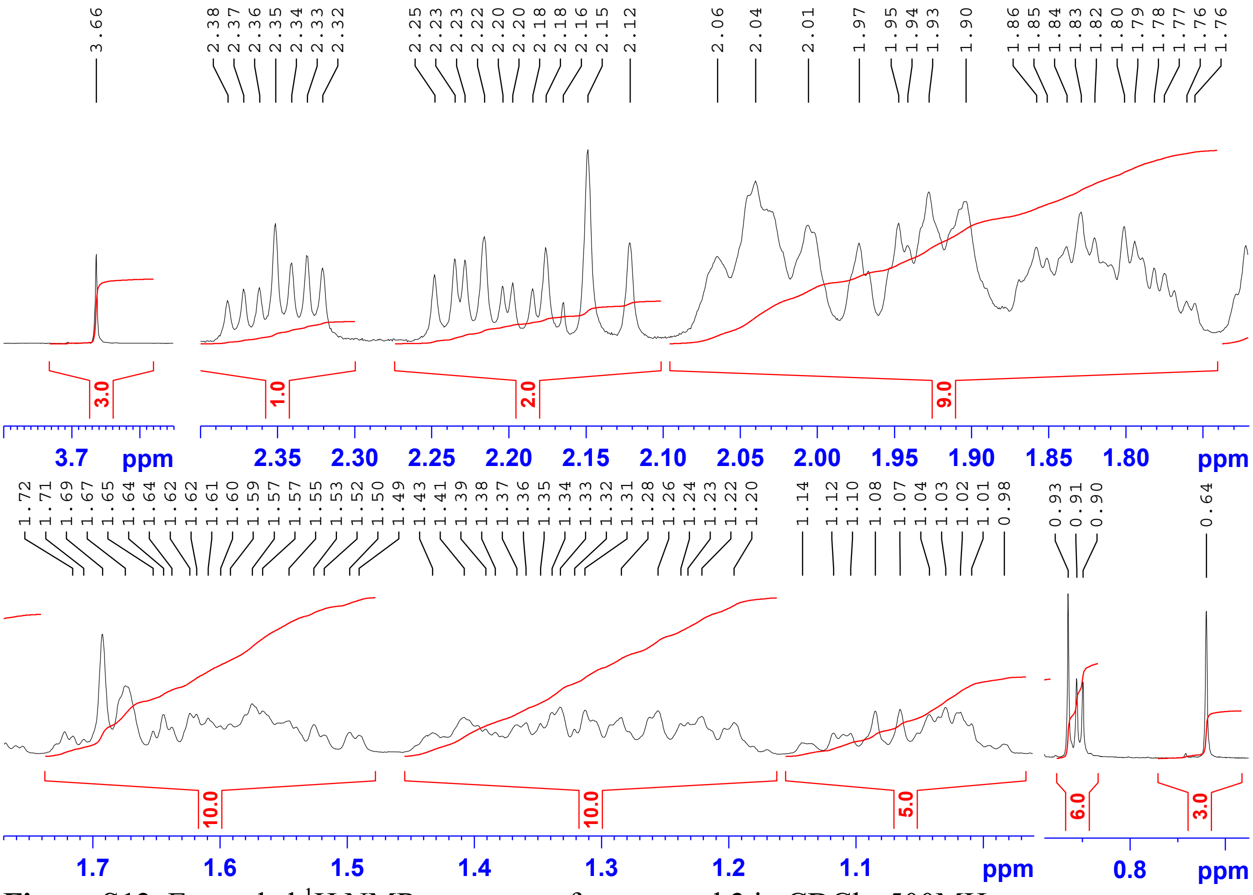

Figure S12. Expanded ^{1}H NMR spectrum of compound **3** in $CDCl_3$, 500MHz.

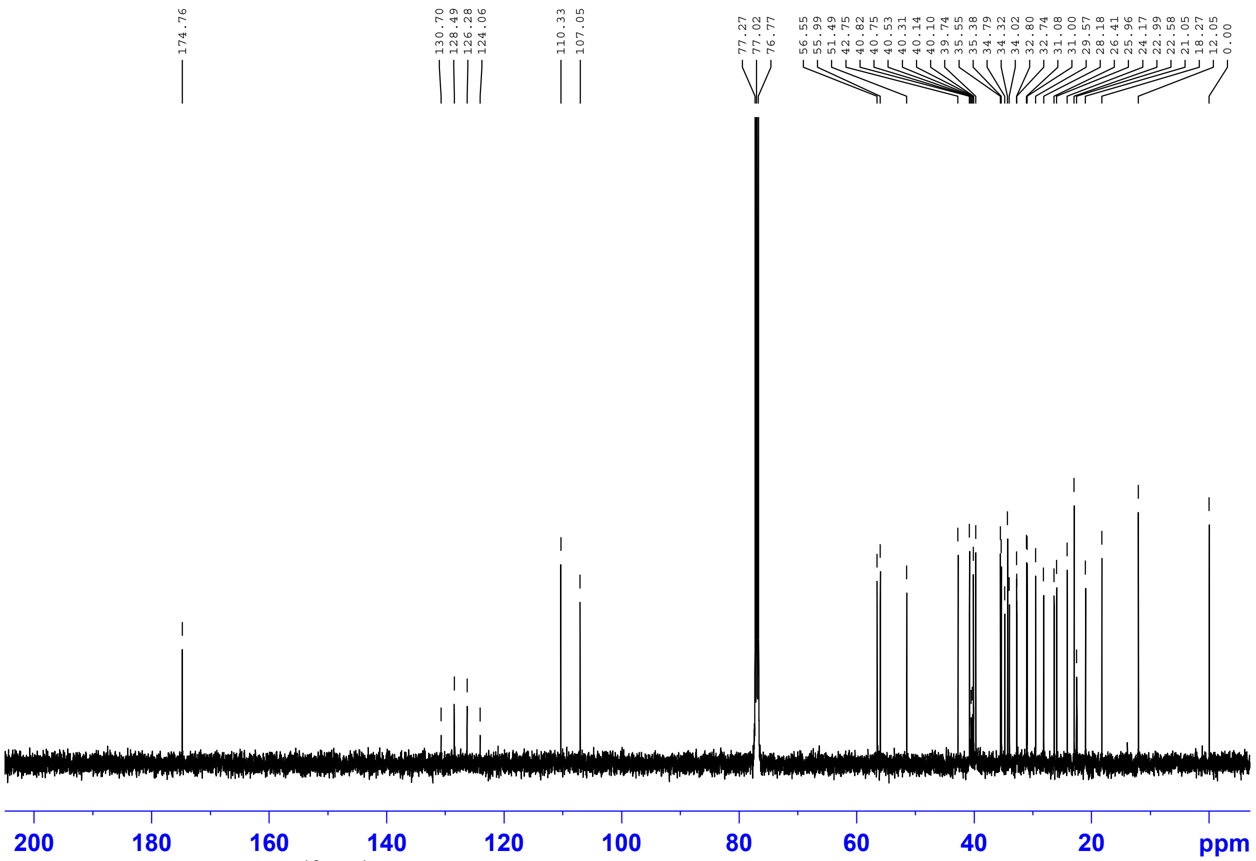

Figure S13. Complete $^{13}C\{^{1}H\}$ spectrum of compound **3** in $CDCl_3$, 125 MHz.

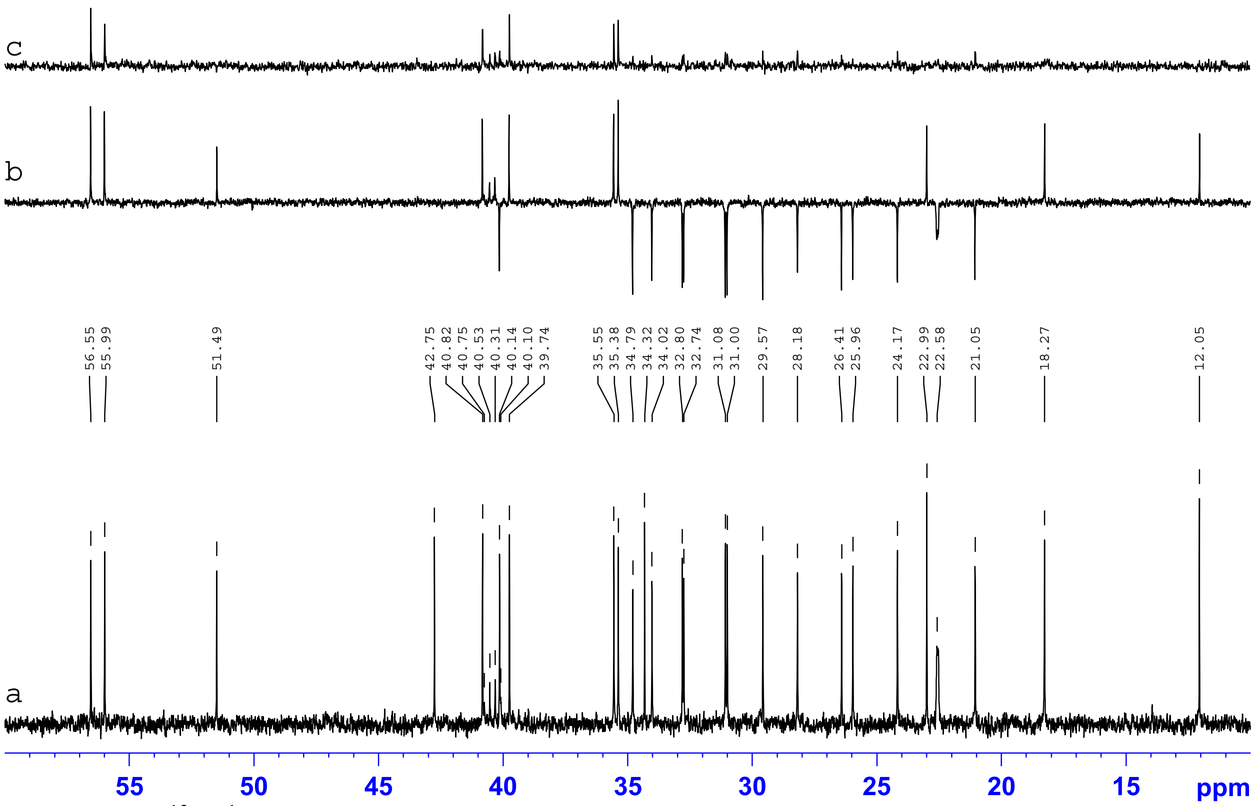

Figure S14. $^{13}C\{^{1}H\}$(a) NMR, DEPT-135 (b) and DEPT-90(c) spectra of compound **3** in $CDCl_3$, 125 MHz.

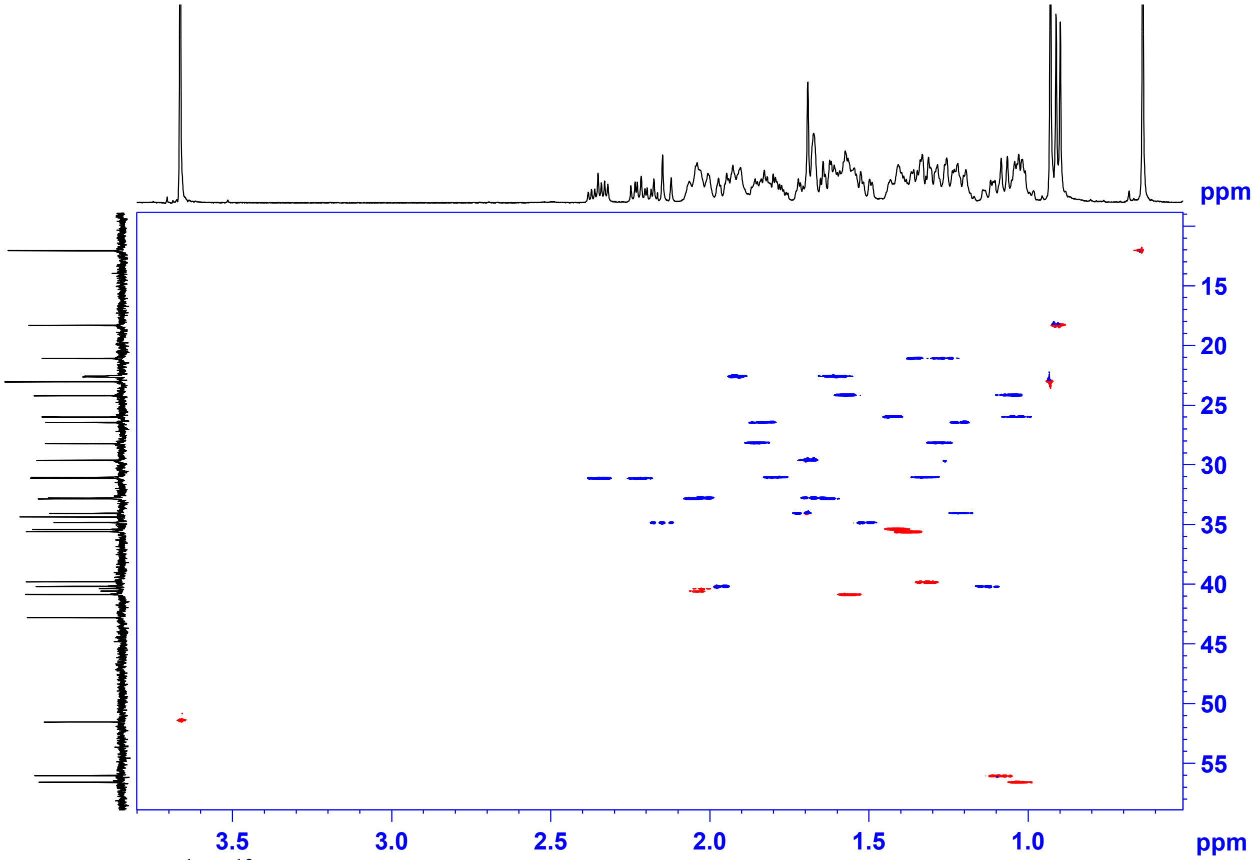

Figure S15. {^{1}H, ^{13}C} HSQCed spectrum of compound **3** in $CDCl_3$, 500 MHz.

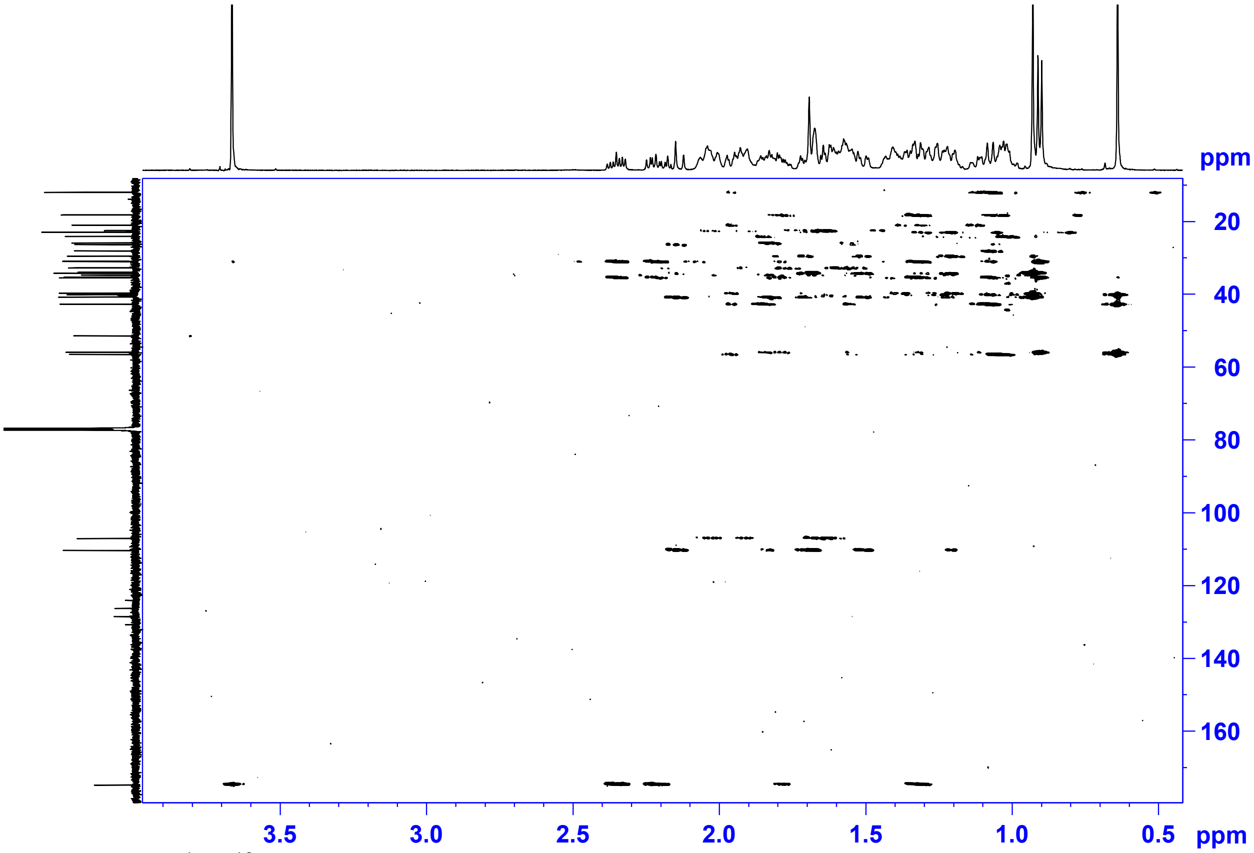

Figure S16. {^{1}H, ^{13}C} HMBC spectrum of compound **3** in $CDCl_3$, 500 MHz.

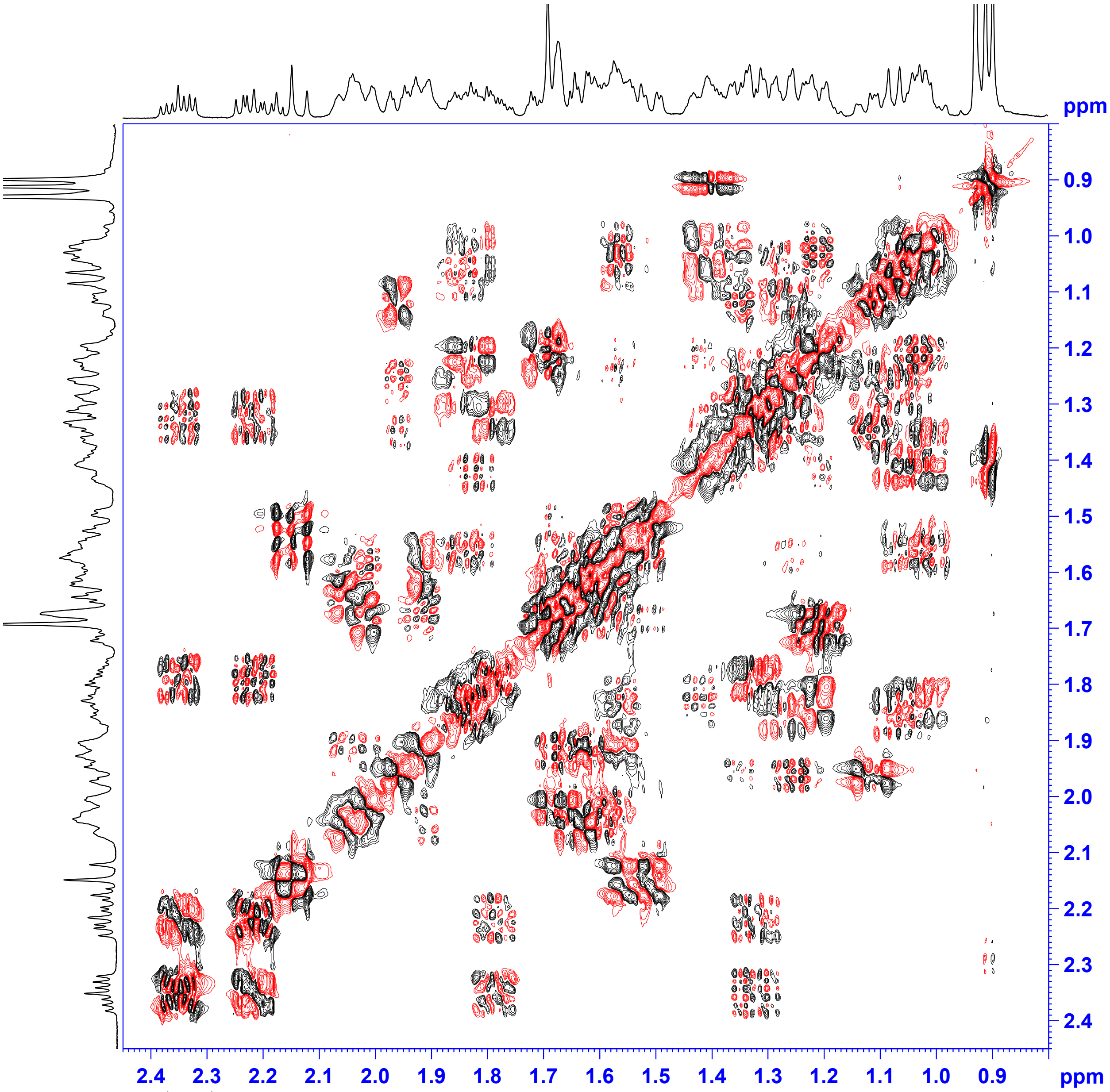

Figure S17. {^1H, ^1H} COSY-DQF spectrum of compound **3** in $CDCl_3$, 500 MHz.

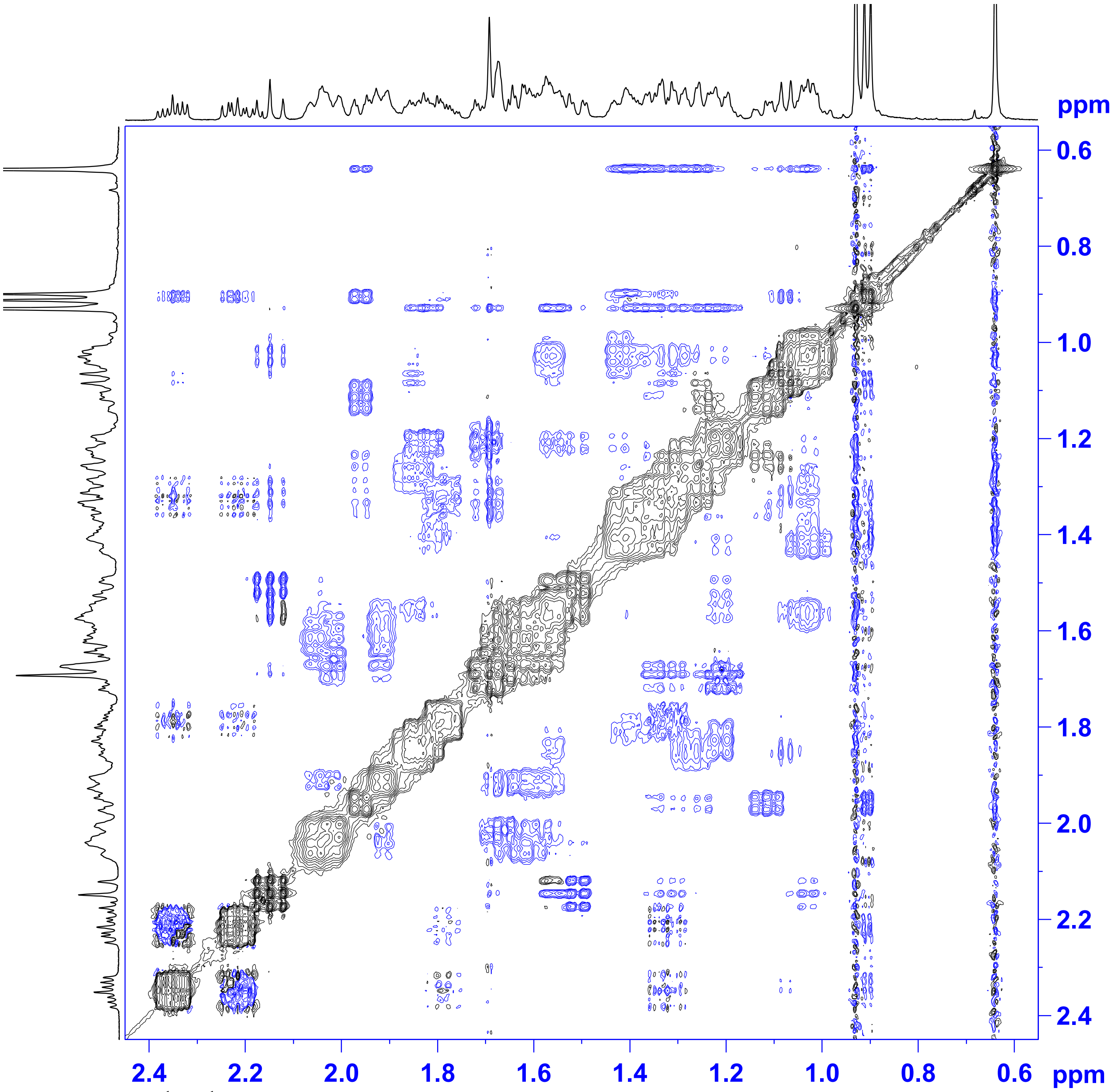

Figure S18. {^{1}H, ^{1}H} NOESY spectrum of compound **3** in $CDCl_3$, 500 MHz.

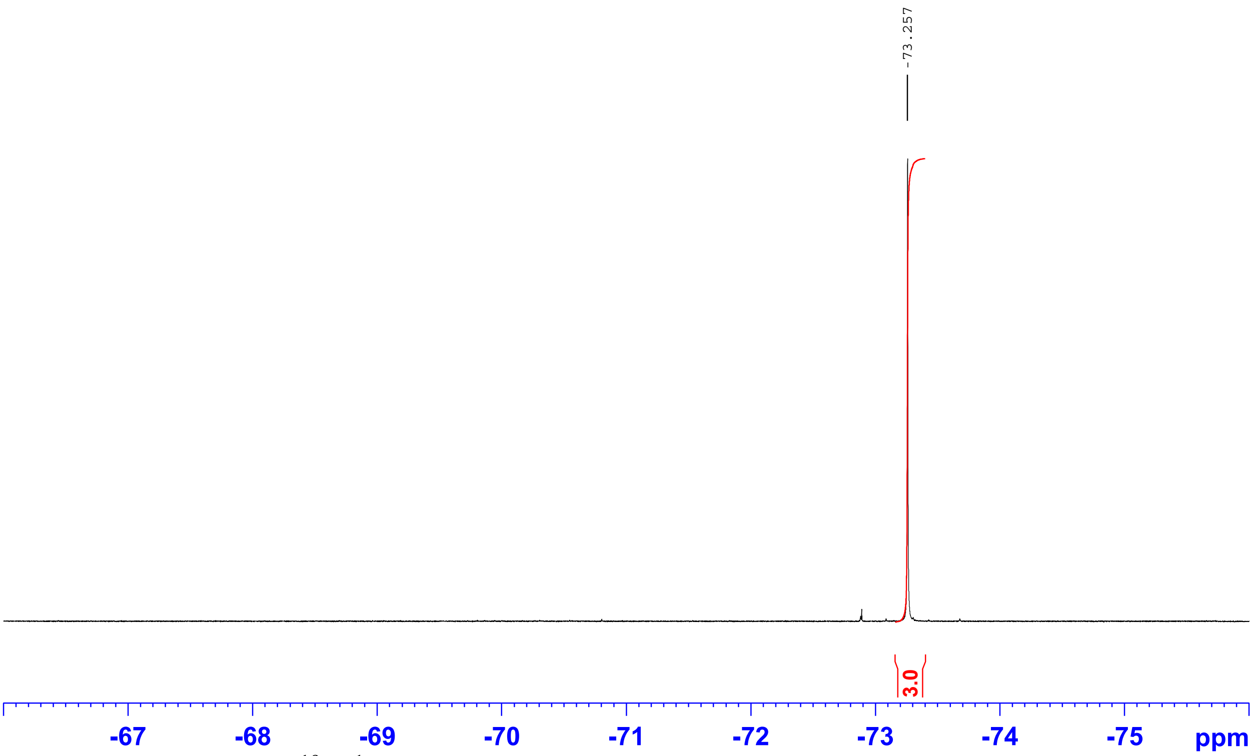

Figure S19. Complete $^{19}F\{^{1}H\}$ NMR spectrum of compound **3** in $CDCl_3$, 470MHz.

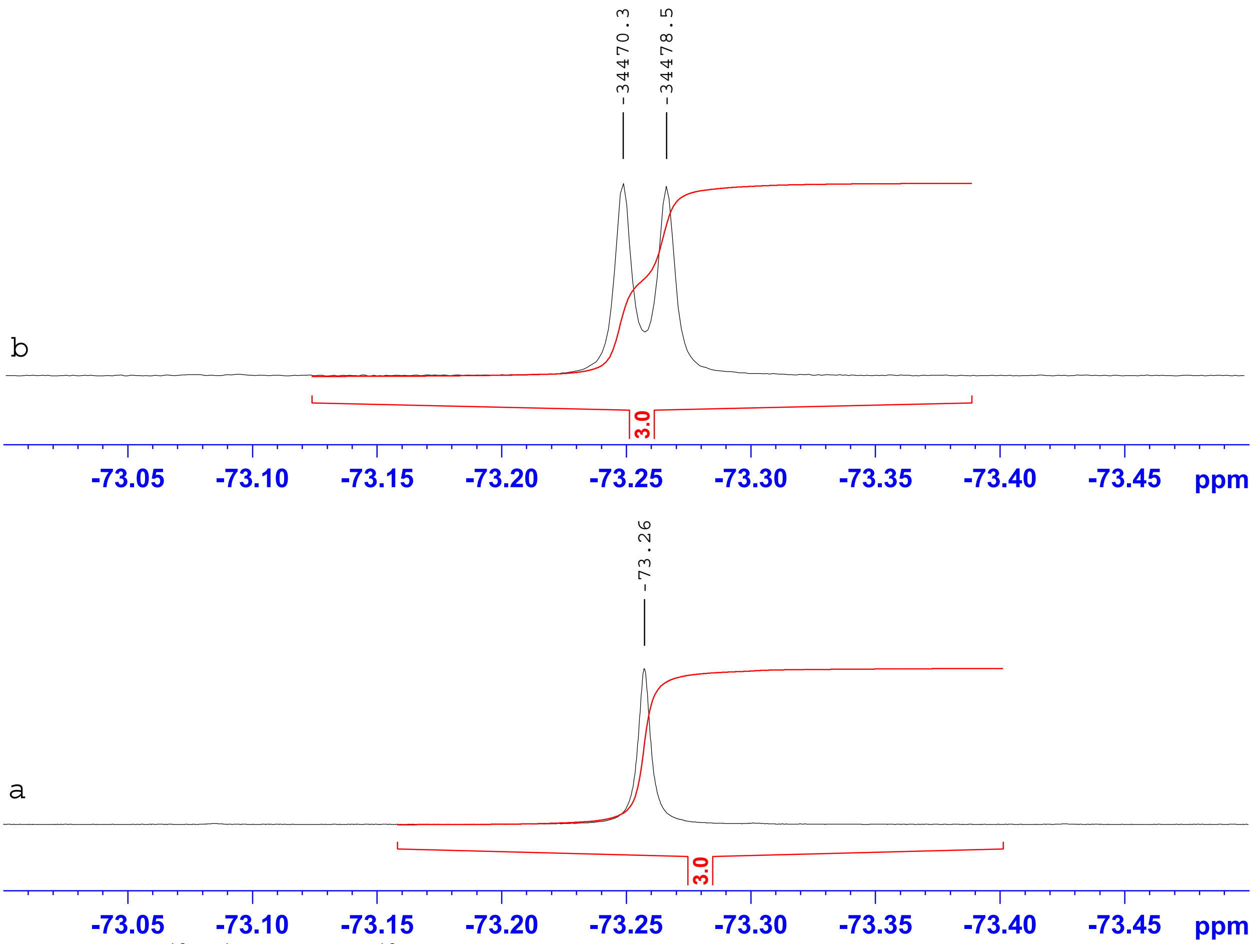

Figure S20. $^{19}F\{^{1}H\}$ (a) and ^{19}F (b) NMR spectra of compound **3** in $CDCl_3$, 470MHz.

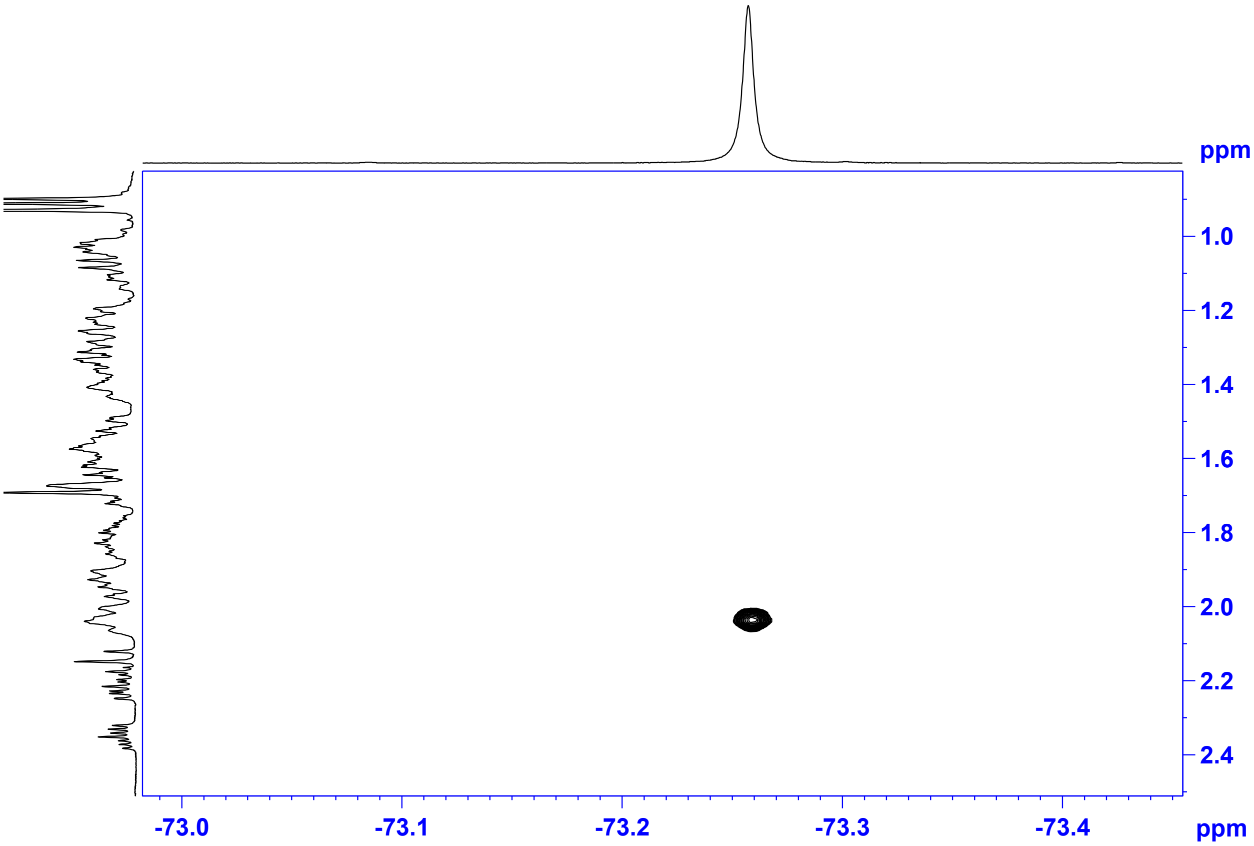

Figure S21. {^{19}F, ^{1}H} HETCOR spectrum of compound **3** in CDCl3, 470MHz.

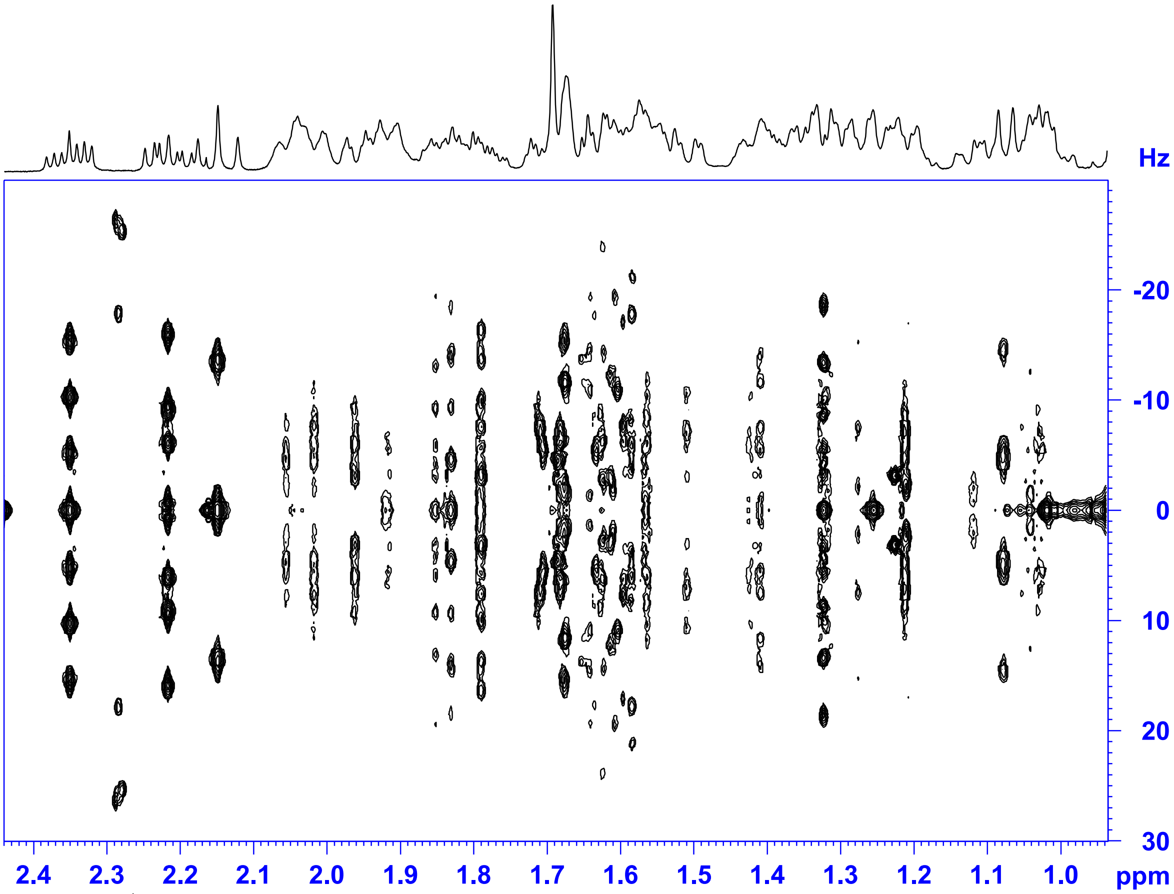

Figure S22. ^{1}H *J*-RES spectrum of compound **3** in CDCl3, 500 MHz.

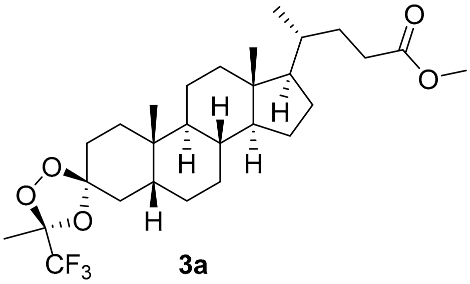

3a

Figure S23. The structure of compound **3a** published in [Yamansarov E. Y. et al. Synthesis and antimalarial activity of 3′-trifluoromethylated 1, 2, 4-trioxolanes and 1, 2, 4, 5-tetraoxane based on deoxycholic acid //Steroids. – 2018. – T. 129. – C. 17-23.]

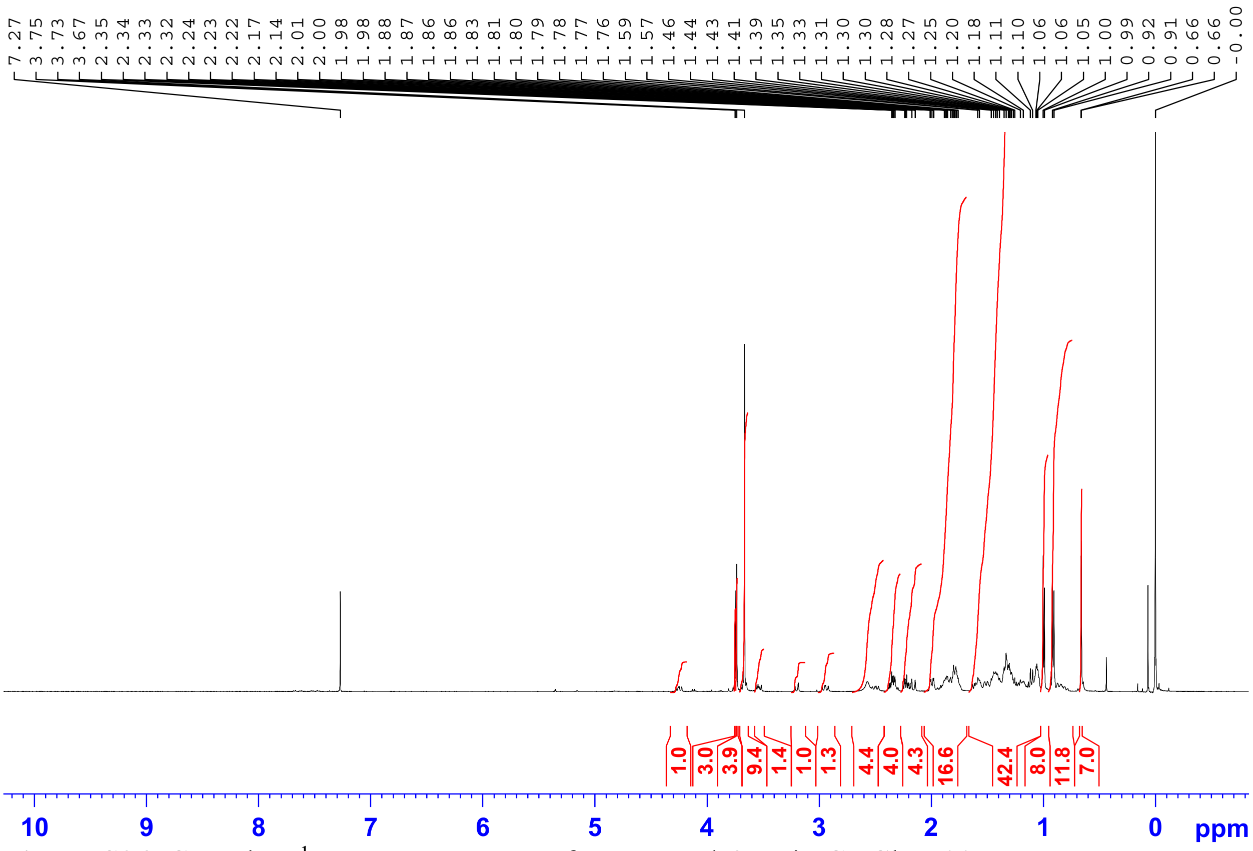

Figure S24. Complete ^{1}H NMR spectrum of compound **4a,b** in CDCl3, 500MHz.

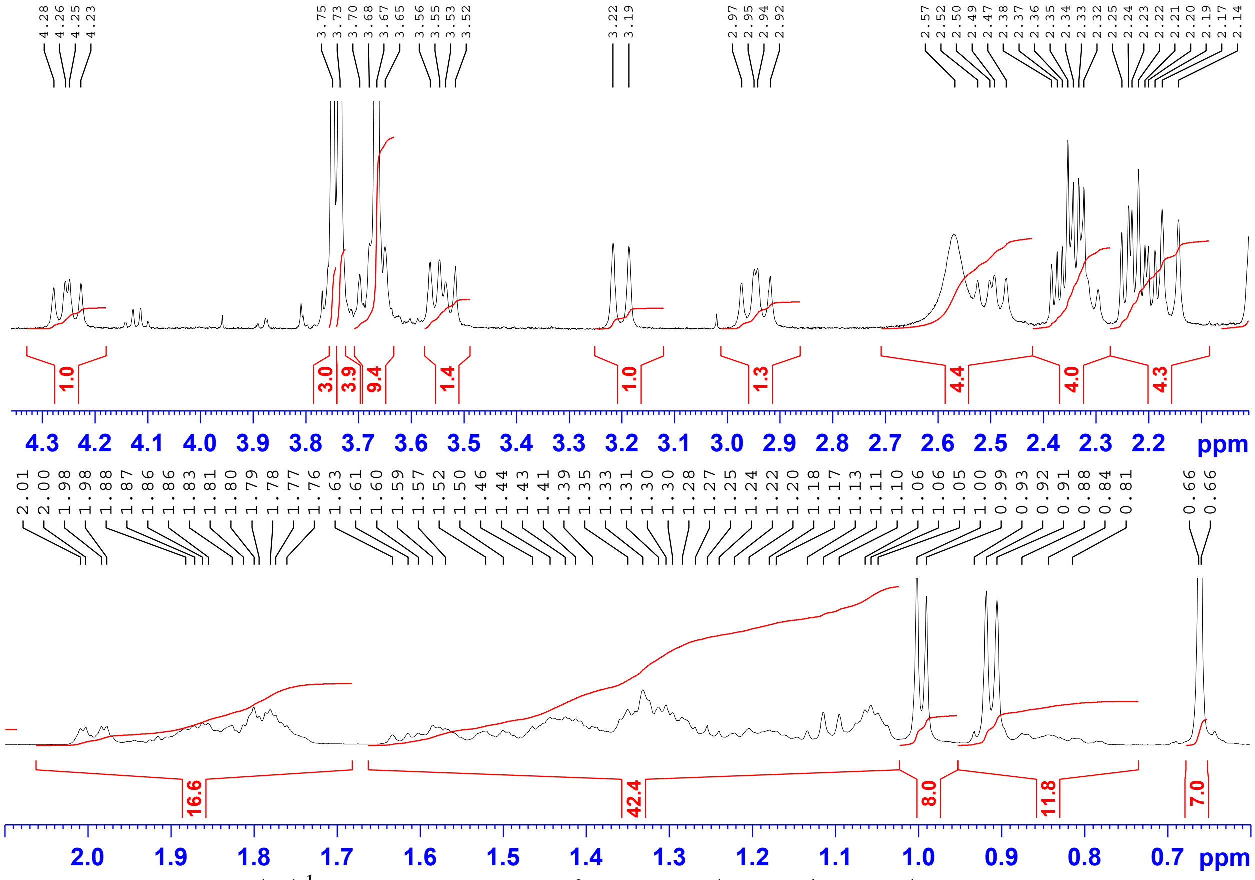

Figure S25. Expanded ^{1}H NMR spectrum of compounds **4a,b** in $CDCl_3$, 500MHz.

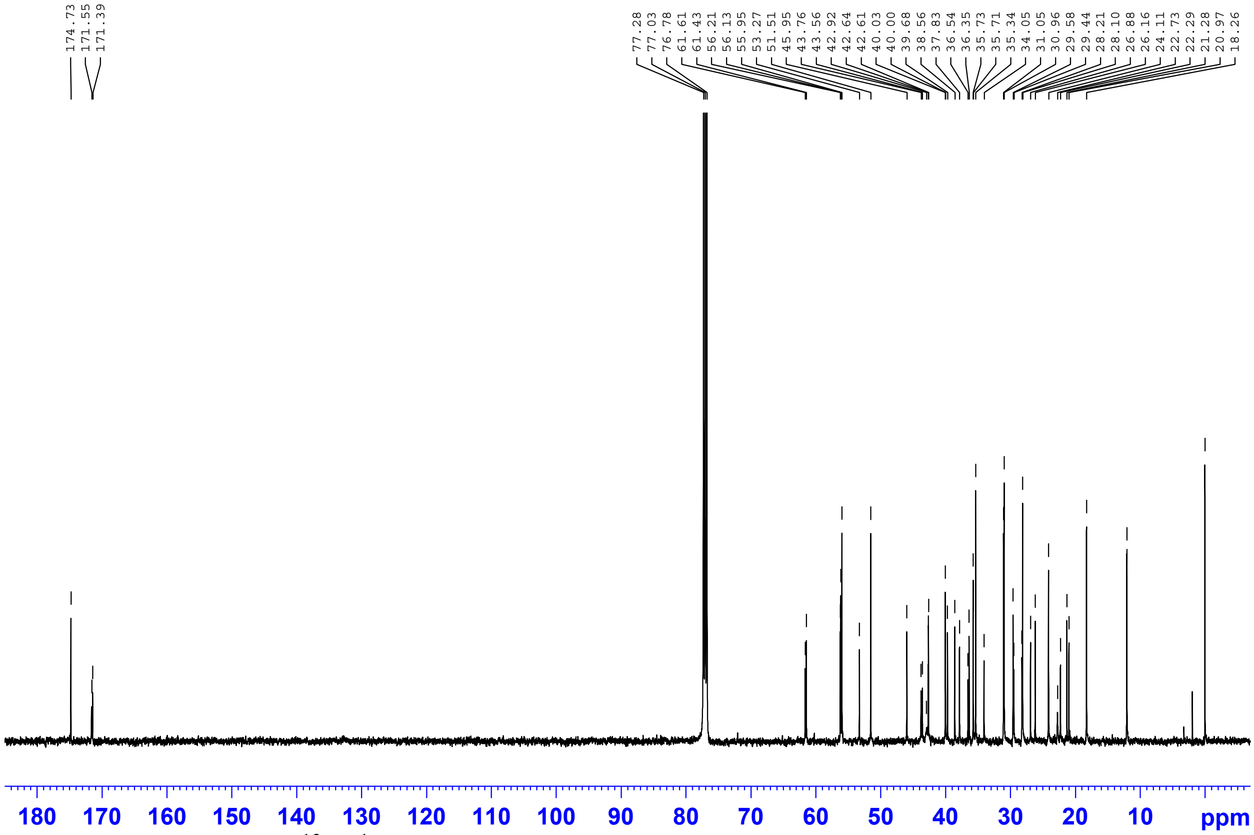

Figure S26. Complete $^{13}C\{^1H\}$ spectrum of compounds **4a,b** in $CDCl_3$, 125 MHz.

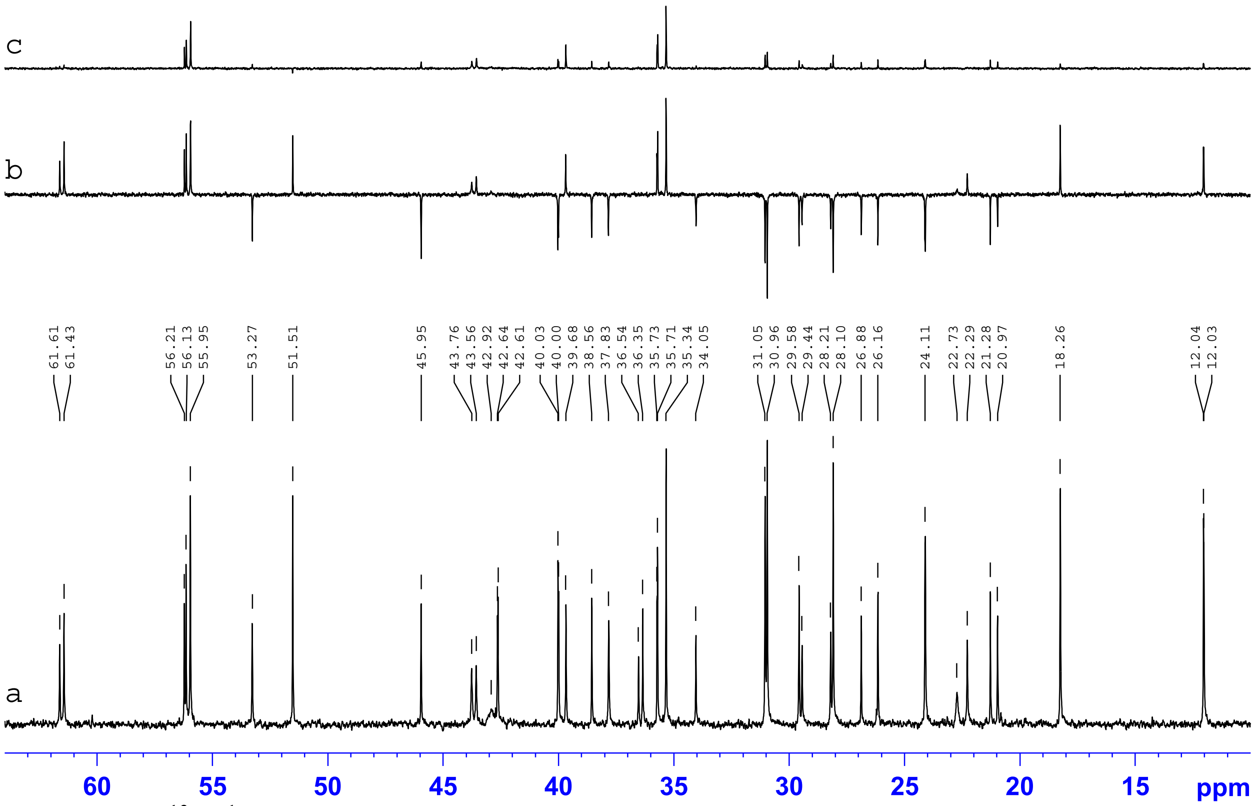

Figure S27. $^{13}C\{^1H\}$(a) NMR, DEPT-135 (b) and DEPT-90(c) spectra of compounds **4a,b** in $CDCl_3$, 125 MHz.

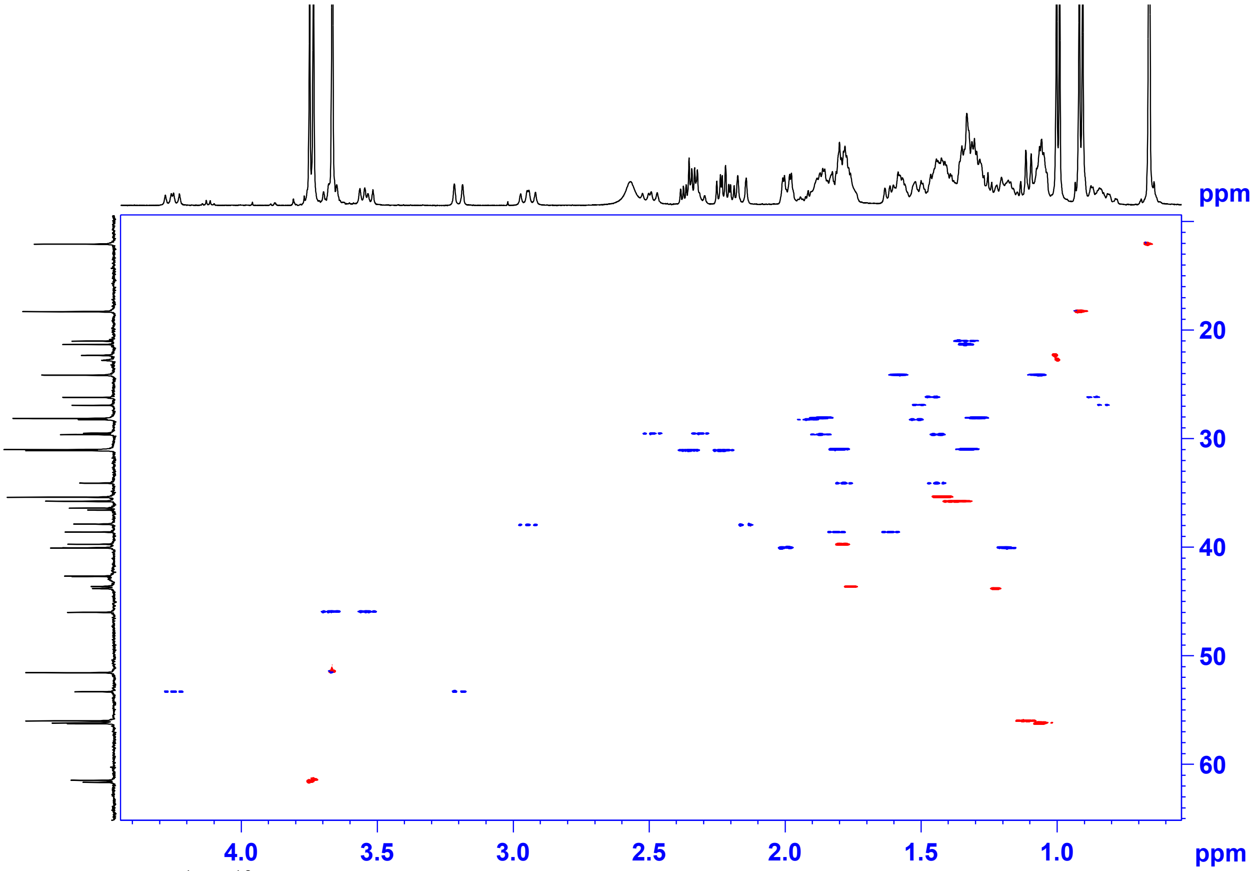

Figure S28. {^{1}H, ^{13}C} HSQCed spectrum of compounds **4a,b** in CDCl3, 500 MHz.

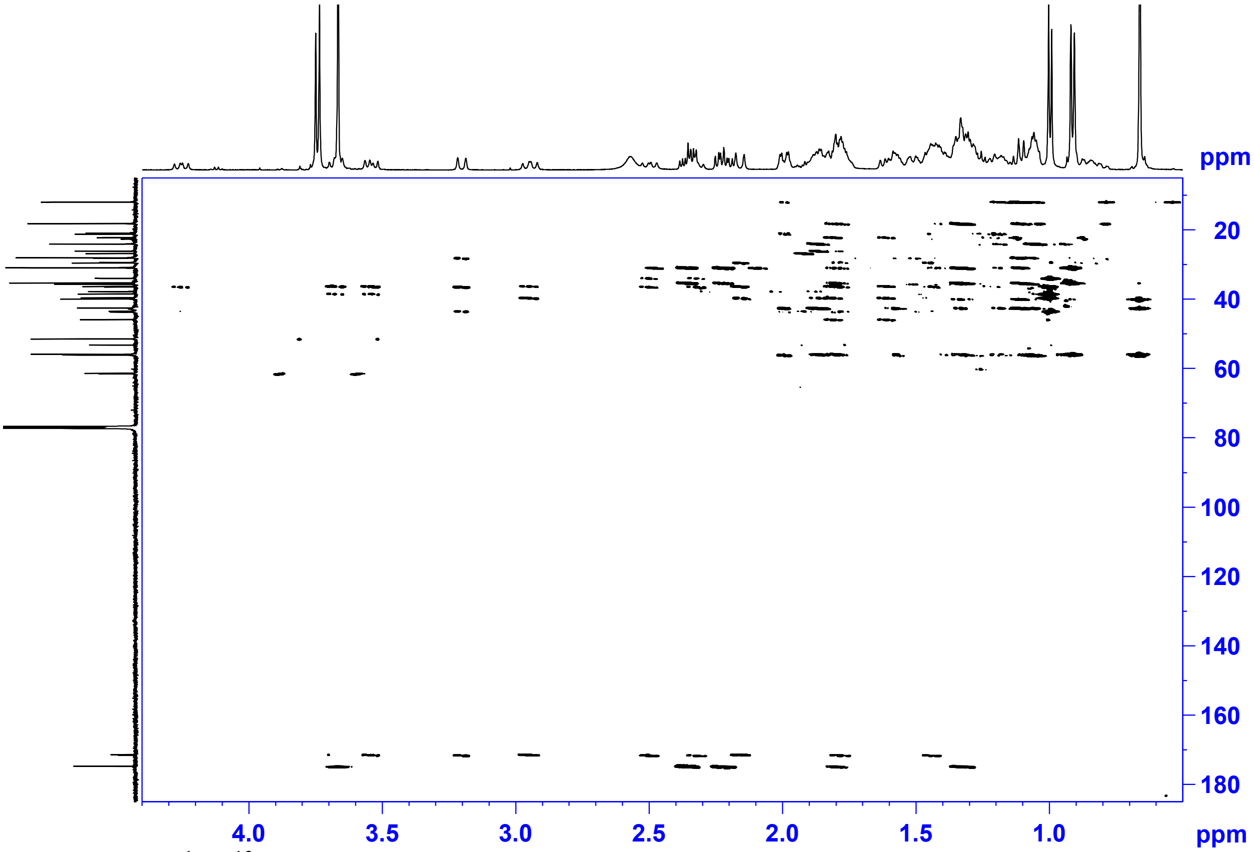

Figure S29. {^{1}H, ^{13}C} HMBC spectrum of compound **4a,b** in CDCl3, 500 MHz.

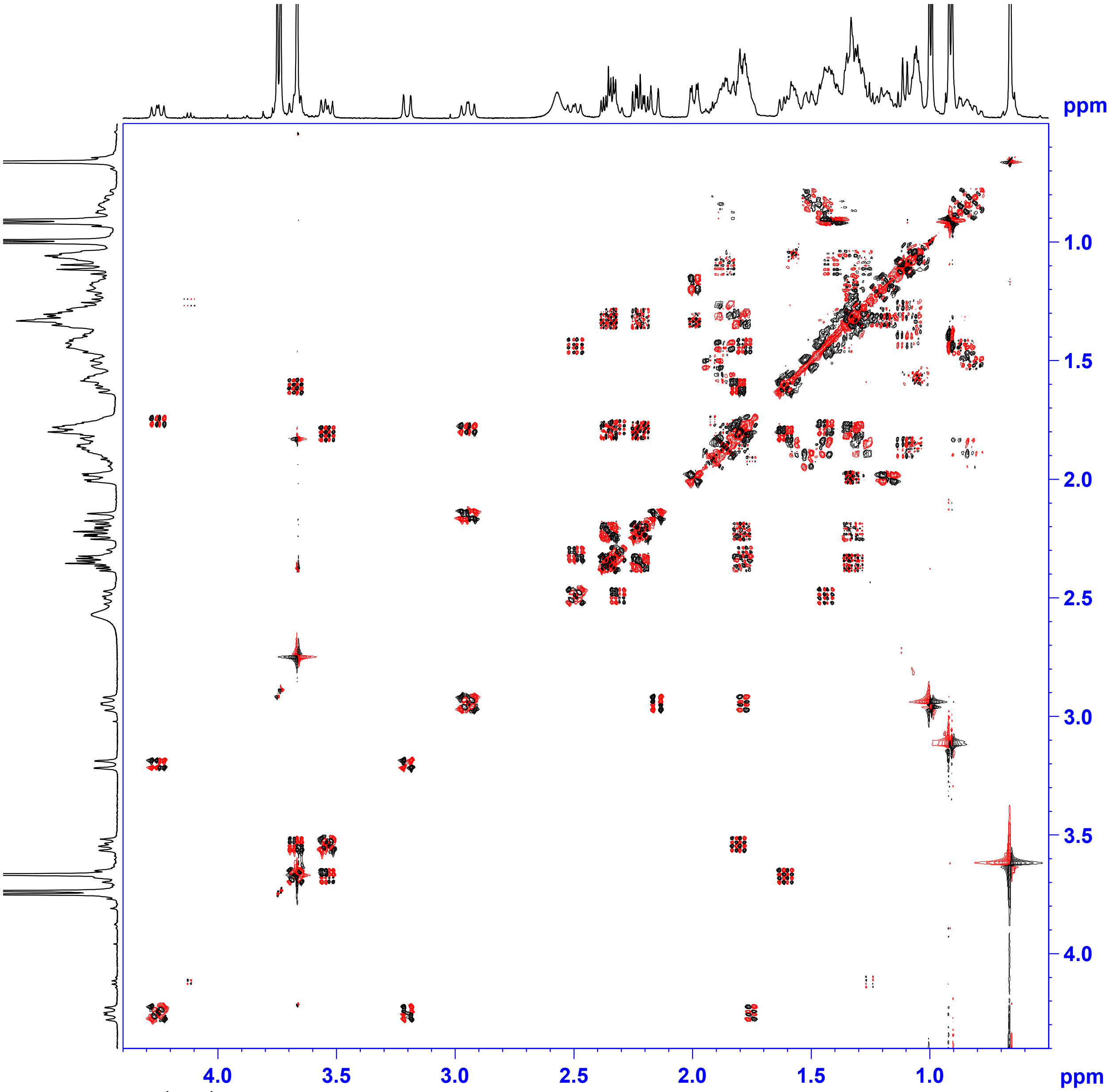

Figure S30. {^{1}H, ^{1}H} COSY-DQF spectrum of compounds **4a,b** in $CDCl_3$, 500 MHz.

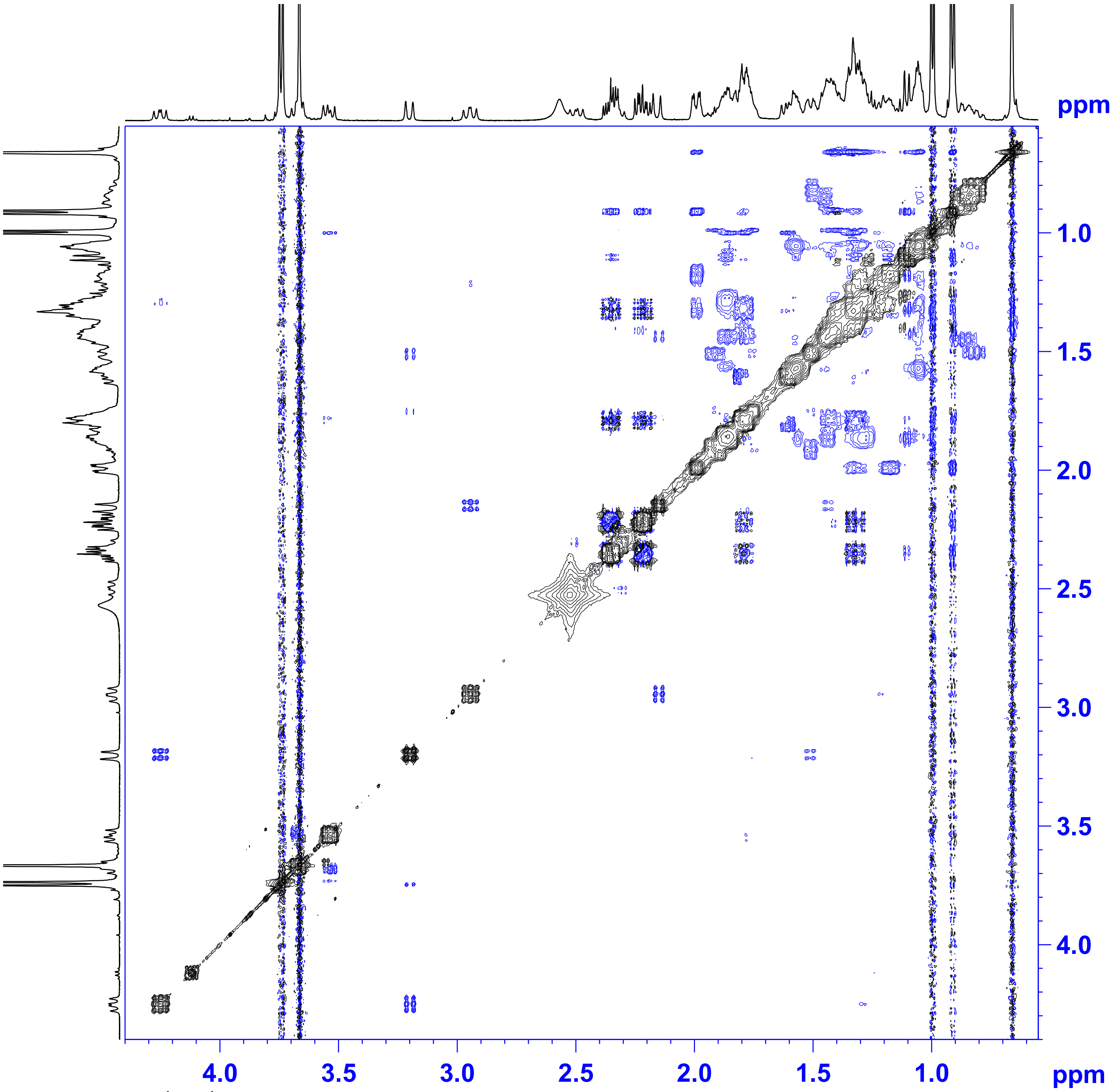

Figure S31. {^{1}H, ^{1}H} NOESY spectrum of compounds **4a,b** in $CDCl_3$, 500 MHz.

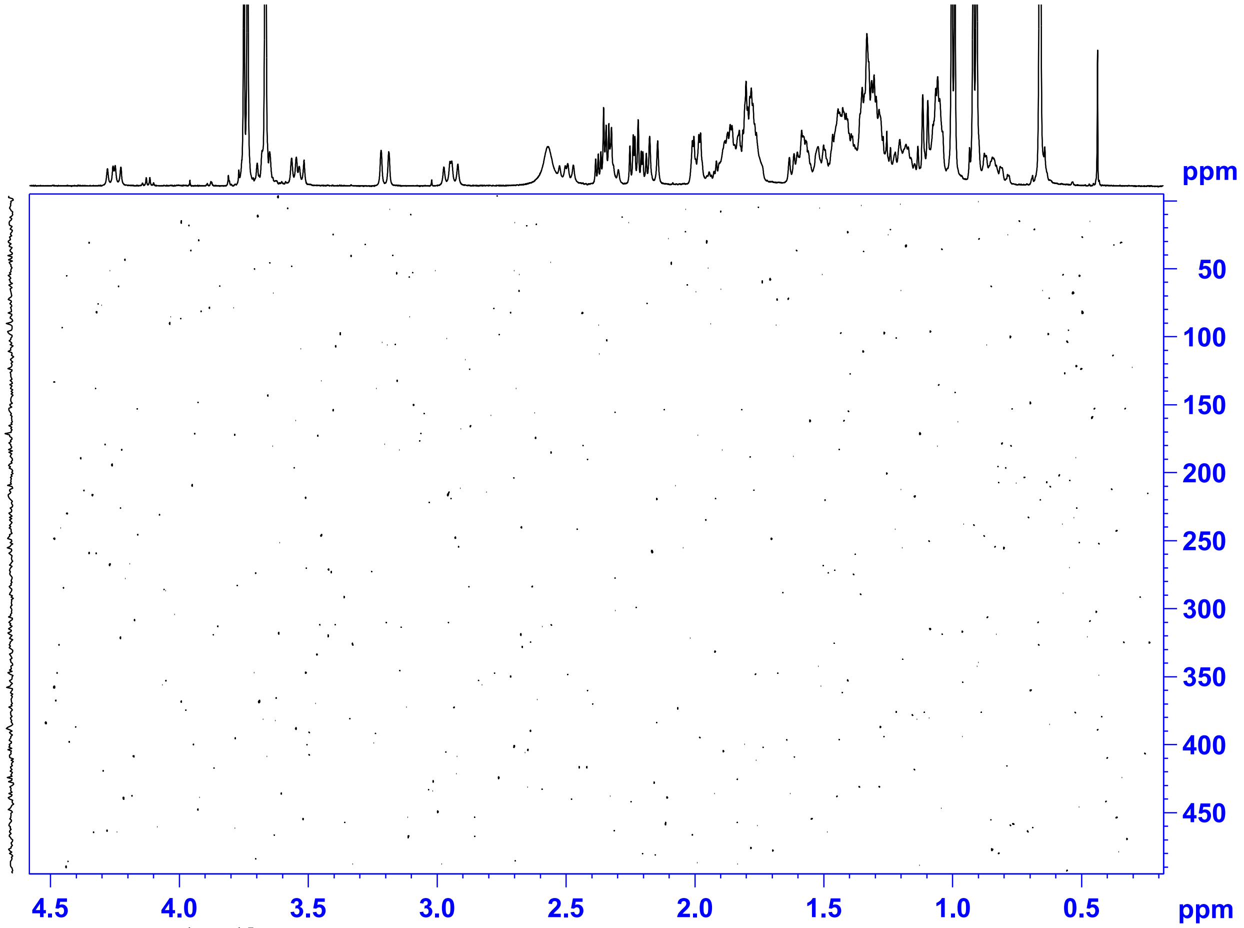

Figure S32. {^{1}H, ^{15}N} HSQC spectrum of compound **4a,b** in $CDCl_3$, 500 MHz.

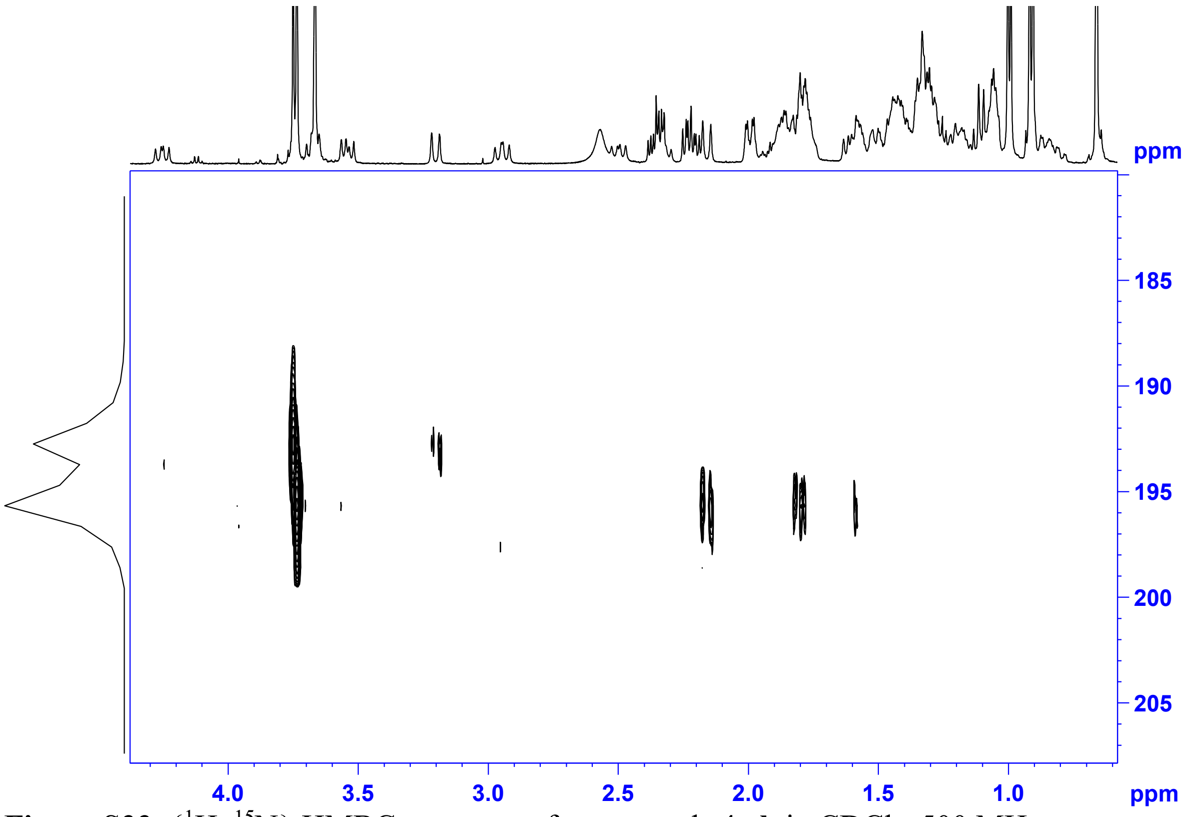

Figure S33. {^{1}H, ^{15}N} HMBC spectrum of compounds **4a,b** in $CDCl_3$, 500 MHz.

Table S1. Crystal data and structure refinement for compound **3**

Empirical formula	$C_{32}H_{49}O_5F_3$
Formula weight	570.71
Temperature/K	296(2)
Crystal system	orthorhombic
Space group	$P2_12_12_1$
a/Å	6.5883(12)
b/Å	22.318(4)
c/Å	42.214(8)
α/°	90
β/°	90
γ/°	90
Volume/Å^3	6207(2)
Z	8
ρ_{calc}g/cm^3	1.221
μ/mm^{-1}	0.092
F(000)	2464.0
Crystal size/mm^3	0.9 × 0.08 × 0.06
Radiation	MoKα (λ = 0.71073)
2Θ range for data collection/°	3.65 to 50.054
Index ranges	$-7 \leq h \leq 4$, $-18 \leq k \leq 26$, $-49 \leq l \leq 50$
Reflections collected	25040
Independent reflections	10933 [R_{int} = 0.0803, R_{sigma} = 0.1261]
Data/restraints/parameters	10933/24/721
Goodness-of-fit on F^2	1.058
Final R indexes [I>=2σ (I)]	R_1 = 0.0909, wR_2 = 0.1595
Final R indexes [all data]	R_1 = 0.1721, wR_2 = 0.1856
Largest diff. peak/hole / e Å^{-3}	0.26/-0.26
Flack parameter	2.5(8)

***In silico* ADMET study and physicochemical, physicochemical profiles of compounds 1 and 3**

To assess the pharmaceutical relevance of the studied molecules, a comparative analysis was conducted for compounds **1** and **3** across key drug development parameters [1], including physicochemical descriptors, lipophilicity, solubility, pharmacokinetic predictions, and drug-likeness criteria, using SWISS ADME (https://www.swissadme.ch/). The calculated data are presented in Table S2.

Table S2. The summary of ADME properties for compounds **1** and **3** as detected by Swiss ADME.

Compound	1	3
Physicochemical Properties		
Formula	$C_{25}H_{40}O_3$	$C_{32}H_{49}F_3O_5$
Molecular weight	388.58 g/mol	570.72 g/mol
Num. heavy atoms	28	40
Num. arom. heavy atoms	0	0
Fraction Csp3	0.92	0.97
Num. rotatable bonds	5	6
Num. H-bond acceptors	3	8
Num. H-bond donors	0	0
Molar Refractivity	114.80	147.54
TPSA	43.37 Å²	53.99 Å²
Lipophilicity		
Log $P_{o/w}$ (iLOGP)	4.12	5.65
Log $P_{o/w}$ (XLOGP3)	6.17	9.27
Log $P_{o/w}$ (WLOGP)	5.80	9.62
Log $P_{o/w}$ (MLOGP)	4.83	6.53
Log $P_{o/w}$ (SILICOS-IT)	5.45	6.66
Consensus Log $P_{o/w}$	5.27	7.54
Water Solubility		

Log *S* (ESOL)	-5.81	5.65
Solubility	6.07e-04 mg/ml ; 1.56e-06 mol/l	9.27
Class	Moderately soluble	9.62
Log *S* (Ali)	-6.86	6.53
Solubility	5.31e-05 mg/ml ; 1.37e-07 mol/l	6.66
Class	Poorly soluble	7.54
Log *S* (SILICOS-IT)	-5.34	-8.82
Solubility	1.79e-03 mg/ml ; 4.61e-06 mol/l	8.59e-07 mg/ml ; 1.50e-09 mol/l
Class	Moderately soluble	Poorly soluble
Pharmacokinetics		
GI absorption	High	Low
BBB permeant	Yes	No
P-gp substrate	No	No
CYP1A2 inhibitor	No	No
CYP2C19 inhibitor	No	Yes
CYP2C9 inhibitor	Yes	No
CYP2D6 inhibitor	No	No
CYP3A4 inhibitor	No	No
Log K_p (skin permeation)	-4.29 cm/s	-3.20 cm/s
Druglikeness		
Lipinski	Yes; 1 violation: MLOGP>4.15	No; 2 violations: MW>500, MLOGP>4.15
Ghose	No; 1 violation: WLOGP>5.6	No; 4 violations: MW>480, WLOGP>5.6, MR>130, #atoms>70

Veber	Yes	Yes
Egan	Yes	No; 1 violation: WLOGP>5.88
Muegge	No; 1 violation: XLOGP3>5	No; 1 violation: XLOGP3>5
Bioavailability Score	0.55	0.17
Medicinal Chemistry		
PAINS	0 alert	0 alert
Brenk	0 alert	1 alert: peroxide
Leadlikeness	No; 2 violations: MW>350, XLOGP3>3.5	No; 2 violations: MW>350, XLOGP3>3.5
Synthetic accessibility	4.69	7.77

Physicochemical Profile

Compound **1** ($C_{25}H_{40}O_3$; MW = 388.58 g/mol) is a medium-sized, highly saturated molecule with 28 heavy atoms and no aromatic components. In contrast, compound **3** ($C_{32}H_{49}F_3O_5$) has a substantially higher molecular weight (570.72 g/mol) and complexity (40 heavy atoms), along with increased hydrogen bond acceptors (8 vs. 3) and molar refractivity (147.54 vs. 114.80), indicating higher polarizability and steric bulk. Both compounds display high sp^3 character (fraction $Csp^3 > 0.9$), a feature associated with improved clinical success rates due to enhanced three-dimensionality and target specificity [2].

Lipophilicity and Solubility

Lipophilicity, a key determinant of membrane permeability and solubility, was predicted using multiple algorithms. Compound **3** exhibits markedly higher lipophilicity across all models, with a consensus LogP of 7.54, exceeding the optimal range for oral drugs (LogP < 5) [3]. Compound **1** shows a more balanced profile (LogP = 5.27), albeit still approaching the upper limit. As expected, this difference significantly impacts aqueous solubility: compound **1** is predicted to be moderately soluble, whereas compound **3** is classified as poorly soluble, with logS values below –6.5 in all models. High lipophilicity and poor solubility are known contributors to poor oral bioavailability and erratic pharmacokinetics [4].

Pharmacokinetic Predictions

In silico ADME analysis indicates that compound **1** has high gastrointestinal (GI) absorption and is blood–brain barrier (BBB) permeant, enhancing its potential for central and peripheral activity. Conversely, compound **3** is predicted to have low GI ab-sorption and is not BBB permeant, likely due to its larger size and poor solubility. Additionally, compound **1** is predicted to inhibit CYP2C9, while compound 3 shows no major cytochrome P450 interactions. Notably, skin permeation (log Kp) is higher for compound **3** (–3.20 cm/s), reflecting its greater lipophilicity.

Druglikeness and Medicinal Chemistry Filters

Compound **1** satisfies most classical druglikeness filters, with only one Lipinski violation (MLOGP > 4.15). In contrast, compound **3** fails two Lipinski criteria (MW > 500 and MLOGP > 4.15), in addition to violations in the Ghose and Egan filters. The bioavailability score is also significantly more favorable for compound 1 (0.55 vs. 0.17) [5]. Compound **3** prompts a Brenk alert for a peroxide group, which may raise con-cerns regarding chemical stability and toxicity [6]. These parameters are presented at Fig. S34.

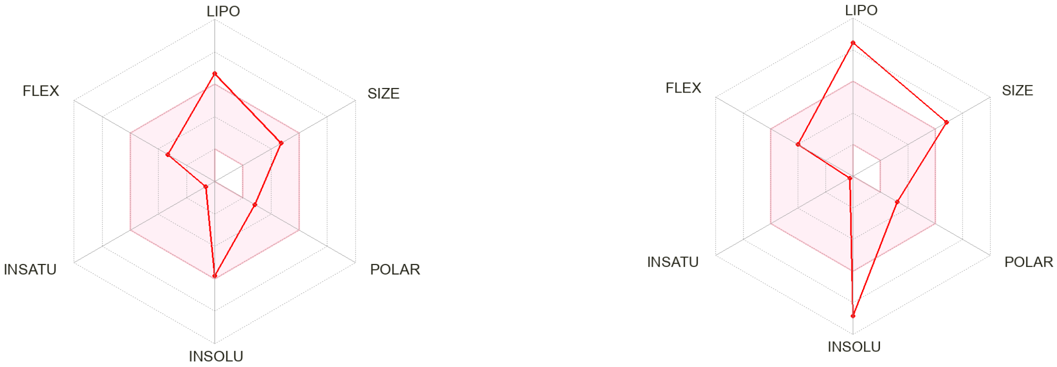

(**a**) (**b**)

Figure S34. The Bioavailability Radar (a) for compound **1**, (b) for compound **3**

Despite its predicted limited aqueous solubility and poor oral bioavailability, compound **3** exhibits a combination of structural and physicochemical properties that may offer distinct therapeutic advantages, particularly in the context of antimalarial drug development. Among its notable features is a high fraction of sp^3-hybridized carbon atoms ($Fsp^3 = 0.97$), which reflects a three-dimensional structure often associated with improved target selectivity, reduced off-target interactions, and enhanced clinical progression [2]. In addition, compound **3** contains eight hydrogen bond acceptors, allowing for increased potential to engage in polar interactions with protein targets, which may enhance binding affinity and specificity [7].

Although highly lipophilic (consensus LogP = 7.54), this characteristic can support passive diffusion across lipid-rich membranes, which is particularly relevant for intracellular parasites like P. falciparum [8]. Furthermore, compound **3** demonstrates a relatively favorable predicted skin permeability (Log Kp = –3.20 cm/s), suggesting potential use in topical or transdermal applications [9]. The trifluoromethyl-substituent contributes to increased metabolic stability, modulates the electronic environment, and may promote halogen bonding with enzyme active sites — features commonly lever-aged in modern drug design [10, 11]. Trifluoroketones are characterized by increased reactivity, in addition, the CF_3 group is a well-known pharmacophore, contained, for example, in the structure of the widely used antimalarial agent mefloquine [12]. Nota-bly, the molecule also contains a peroxide moiety, a pharmacophore central to the mechanism of action of antimalarial drugs such as artemisinin. Although flagged by structural filters (e.g., Brenk alerts), the inclusion of this redox-active group may support mechanism-based antiparasitic activity [6].

References

1. Daina, A.; Michielin, O.; Zoete, V. SwissADME: A Free Web Tool to Evaluate Pharmacokinetics, Druglikeness and Medicinal Chemistry Friendliness of Small Molecules. *Sci. Rep.* **2017**, *7*, 42717. https://doi.org/10.1038/srep42717
2. Lovering, F.; Bikker, J.; Humblet, C. Escape from Flatland: Increasing Saturation as an Approach to Improving Clinical Success. *J. Med. Chem.* **2009**, *52*, 6752–6756. https://doi.org/10.1021/jm901241e
3. Lipinski, C.A. Lead- and Drug-Like Compounds: The Rule-of-Five Revolution. *Drug Discov. Today Technol.* **2004**, *1*, 337–341. https://doi.org/10.1016/j.ddtec.2004.11.007
4. Di, L.; Fish, P.V.; Mano, T. Bridging Solubility between Drug Discovery and Development. *Drug Discov. Today* **2012**, *17*, 486–495. https://doi.org/10.1016/j.drudis.2012.01.013
5. Martin, Y.C. A Bioavailability Score. *J. Med. Chem.* **2005**, *48*, 3164–3170. https://doi.org/10.1021/jm0492002
6. Brenk, R.; Schipani, A.; James, D.; Krasowski, A.; Gilbert, I.H.; Frearson, J. Lessons Learnt from Assembling Screening Libraries for Neglected Diseases. *ChemMedChem* **2008**, *3*, 435–444. https://doi.org/10.1002/cmdc.200700139
7. Kenny, P.W. Hydrogen-Bond Donors in Drug Design. *J. Med. Chem.* **2022**, *65*, 14261–14275. https://doi.org/10.1021/acs.jmedchem.2c01147
8. Duffy, S.; Avery, V.M. Development and Optimization of a Novel 384-Well Anti-Malarial Imaging Assay. *Malar. J.* **2013**, *12*, 419. https://doi.org/10.1186/1475-2875-12-419
9. Potts, R.O.; Guy, R.H. Predicting Skin Permeability. *Pharm. Res.* **1992**, *9*, 663–669. https://doi.org/10.1023/A:1015810312465
10. Zhang, C. Fluorine in Medicinal Chemistry: In Perspective to COVID-19. *ACS Omega* **2022**, *7*, 18206–18212. https://doi.org/10.1021/acsomega.2c01121
11. Park, B.K.; Kitteringham, N.R.; O'Neill, PM. Metabolism of Fluorine-Containing Drugs. *Annu. Rev. Pharmacol. Toxicol.* **2001**, *41*, 443–470. https://doi.org/10.1146/annurev.pharmtox.41.1.443
12. Upadhyay, C.; Chaudhary, M.; De Oliveira, R.N.; Borbas, A.; Kempaiah, P.; Rathi, B. Fluorinated Scaffolds for Antimalarial Drug Discovery. *Expert Opin. Drug Discov.* **2020**, *15*, 705–718. https://doi.org/10.1080/17460441.2020.1740203
